# Inequalities in Alcohol-Related Mortality in 17 European Countries: A Retrospective Analysis of Mortality Registers

**DOI:** 10.1371/journal.pmed.1001909

**Published:** 2015-12-01

**Authors:** Johan P. Mackenbach, Ivana Kulhánová, Matthias Bopp, Carme Borrell, Patrick Deboosere, Katalin Kovács, Caspar W. N. Looman, Mall Leinsalu, Pia Mäkelä, Pekka Martikainen, Gwenn Menvielle, Maica Rodríguez-Sanz, Jitka Rychtaříková, Rianne de Gelder

**Affiliations:** 1 Department of Public Health, Erasmus University Medical Center, Rotterdam, The Netherlands; 2 Epidemiology, Biostatistics and Prevention Institute, University of Zurich, Zurich, Switzerland; 3 Agència de Salut Pública de Barcelona, Barcelona, Spain; 4 Department of Sociology, Vrije Universiteit Brussel, Brussels, Belgium; 5 Demographic Research Institute, Hungarian Central Statistical Office, Budapest, Hungary; 6 Stockholm Centre for Health and Social Change, Södertörn University, Huddinge, Sweden; 7 Department of Epidemiology and Biostatistics, National Institute for Health Development, Tallinn, Estonia; 8 National Institute for Health and Welfare, Helsinki, Finland; 9 Department of Sociology, University of Helsinki, Helsinki, Finland; 10 Sorbonne Universités, Université Pierre et Marie Curie (Paris 6), INSERM, Institut Pierre Louis d’Epidémiologie et de Santé Publique (UMRS 1136), Paris, France; 11 Department of Demography, Charles University, Prague, Czech Republic; University of Toronto, CANADA

## Abstract

**Background:**

Socioeconomic inequalities in alcohol-related mortality have been documented in several European countries, but it is unknown whether the magnitude of these inequalities differs between countries and whether these inequalities increase or decrease over time.

**Methods and Findings:**

We collected and harmonized data on mortality from four alcohol-related causes (alcoholic psychosis, dependence, and abuse; alcoholic cardiomyopathy; alcoholic liver cirrhosis; and accidental poisoning by alcohol) by age, sex, education level, and occupational class in 20 European populations from 17 different countries, both for a recent period and for previous points in time, using data from mortality registers. Mortality was age-standardized using the European Standard Population, and measures for both relative and absolute inequality between low and high socioeconomic groups (as measured by educational level and occupational class) were calculated.

Rates of alcohol-related mortality are higher in lower educational and occupational groups in all countries. Both relative and absolute inequalities are largest in Eastern Europe, and Finland and Denmark also have very large absolute inequalities in alcohol-related mortality. For example, for educational inequality among Finnish men, the relative index of inequality is 3.6 (95% CI 3.3–4.0) and the slope index of inequality is 112.5 (95% CI 106.2–118.8) deaths per 100,000 person-years. Over time, the relative inequality in alcohol-related mortality has increased in many countries, but the main change is a strong rise of absolute inequality in several countries in Eastern Europe (Hungary, Lithuania, Estonia) and Northern Europe (Finland, Denmark) because of a rapid rise in alcohol-related mortality in lower socioeconomic groups. In some of these countries, alcohol-related causes now account for 10% or more of the socioeconomic inequality in total mortality.

Because our study relies on routinely collected underlying causes of death, it is likely that our results underestimate the true extent of the problem.

**Conclusions:**

Alcohol-related conditions play an important role in generating inequalities in total mortality in many European countries. Countering increases in alcohol-related mortality in lower socioeconomic groups is essential for reducing inequalities in mortality. Studies of why such increases have not occurred in countries like France, Switzerland, Spain, and Italy can help in developing evidence-based policies in other European countries.

## Abstract 2

In a harmonized analysis of regional data, Johan Mackenbach and colleagues characterize three decades of alcohol-related mortality across socioeconomic groups in Europe.

## Editors' Summary

**Background:**

People have consumed alcoholic beverages throughout history, but, globally, about three million people die from alcohol-related causes every year. Alcohol consumption, particularly in higher amounts, is a risk factor for cardiovascular disease (diseases of the heart and/or blood vessels), liver cirrhosis (scarring of the liver), injuries, and many other fatal and nonfatal health problems. Alcohol also affects the well-being and health of people around those who drink, through alcohol-related crime and road traffic crashes. The impact of alcohol use on health depends on the amount of alcohol consumed and on the pattern of drinking. Most guidelines on alcohol consumption recommend that men should regularly consume no more than two alcoholic drinks per day and that women should regularly consume no more than one drink per day (a “drink” is, roughly speaking, a can of beer or a small glass of wine). The guidelines also advise people to avoid binge drinking—the consumption of five or more drinks on a single occasion for men or four or more drinks on a single occasion for women.

**Why Was This Study Done?:**

Like many other behaviors that affect health, alcohol consumption is affected by socioeconomic status (an individual’s economic and social position in relation to others based on income, level of education, and occupation). Thus, in many European countries, the frequency of drinking and the levels of alcohol consumption are greater in higher socioeconomic groups than in lower socioeconomic groups, whereas binge drinking and other problematic forms of alcohol consumption occur more frequently in lower socioeconomic groups. Importantly, higher levels of mortality (death) from alcohol-related conditions have been documented in lower socioeconomic groups than in higher socioeconomic groups in several European countries. Here, the researchers analyze mortality registers to find out whether the magnitude of socioeconomic inequalities in alcohol-related mortality differs among European countries and whether these inequalities have changed over time. Documenting these differences and changes is important because it may help to explain socioeconomic inequalities in alcohol-related mortality and thus inform policies and interventions designed to reduce alcohol-related harm and socioeconomic inequalities in mortality.

**What Did the Researchers Do and Find?:**

The researchers obtained data on deaths from alcoholic psychosis, dependence, and abuse; alcoholic cardiomyopathy (a type of heart disease); alcoholic liver cirrhosis; and accidental alcohol poisoning from the mortality registers of 17 European countries. Using available data on educational level and occupational class, they calculated relative and absolute socioeconomic inequalities in alcohol-related mortality (relative inequality reflects mortality differences between socioeconomic groups in terms of a proportion or percentage; absolute inequality reflects mortality differences between groups in terms of deaths per 100,000 person-years). Rates of alcohol-related mortality were higher in individuals with less education or with manual (as opposed to non-manual) occupations in all 17 countries. Both relative and absolute inequalities were largest in Eastern Europe but Finland and Denmark also had very large absolute inequalities in alcohol-related mortality. For example, among Finnish men, those with the lowest level of education were 3.6 times more likely to die from an alcohol-related cause than those with the highest level of education, and there were 112.5 more deaths per 100,000 person-years among those with the lowest level of education than among those with the highest level of education. The relative inequality in alcohol-related mortality increased over time in many countries. Moreover, the absolute inequality increased markedly in Hungary, Lithuania, Estonia, Finland, and Denmark because of a rapid rise in alcohol-related mortality in lower socioeconomic groups. By contrast, mortality from alcohol-related causes among lower educated men was stable in France, Switzerland, Spain, and Italy.

**What Do These Findings Mean?:**

These findings suggest that alcohol-related conditions are an important contributing factor to the socioeconomic inequality in total mortality in many European countries. Indeed, in some countries (for example, Finland), alcohol-related causes account for 10% or more of the socioeconomic inequality in total mortality among men. The accuracy of these findings is likely to be affected by the use of routinely collected underlying causes of death and by other aspects of the study design. Importantly, however, these findings indicate that to reduce socioeconomic inequalities in mortality, health professionals and governments need to introduce interventions and policies designed to counter recent increases in alcohol-related mortality in lower socioeconomic groups. Further investigation of why such increases have not occurred in some countries may help in the design of these important public health initiatives.

**Additional Information:**

This list of resources contains links that can be accessed when viewing the PDF on a device or via the online version of the article at http://dx.doi.org/10.1371/journal.pmed.1001909.

- The World Health Organization provides detailed information about alcohol, including a fact sheet on the harmful use of alcohol; its Global Status Report on Alcohol and Health 2014 provides country profiles for alcohol consumption, information on the impact of alcohol use on health, and policy responses; the Global Information System on Alcohol and Health provides further information about alcohol, including information on control policies around the world
- The US National Institute on Alcohol Abuse and Alcoholism has information about alcohol and its effects on health; it provides interactive worksheets to help people evaluate their drinking and decide whether and how to make a change
- The US Centers for Disease Control and Prevention provides information on alcohol and public health
- The UK National Health Service Choices website provides detailed information about drinking and alcohol, including information on the risks of drinking too much, tools for calculating alcohol consumption, and personal stories about alcohol use problems
- MedlinePlus provides links to many other resources on alcohol

## Introduction

Alcohol consumption, particularly in higher doses, is a risk factor for many fatal and nonfatal health problems. Acute intoxication is a risk factor for injuries, both accidental and non-accidental, and for cardiovascular disease, whereas chronic heavy drinking is a risk factor for liver cirrhosis, various forms of cancer, and cardiovascular disease [1,2].

Liver cirrhosis mortality is high in Europe, particularly in Central and Eastern European countries [3]. Mortality from liver cirrhosis used to be high in Southern Europe, too, but since the 1970s and 1980s it has declined substantially in France, Italy, Spain, Greece, and other Mediterranean countries. By contrast, mortality from liver cirrhosis has increased in Central and Eastern Europe, in Finland, and in the United Kingdom [3–6].

Patterns and trends of mortality from other alcohol-related conditions have been studied less often. Within Europe, mortality from alcoholic psychosis, alcohol poisoning, and alcohol dependence appears to have a geographical pattern different from that of mortality from liver cirrhosis, with relatively high rates of the former in the Nordic countries [7,8]. This has been attributed to the culture of binge drinking, characterized by non-daily drinking and frequent intoxication, in Northern Europe, which is also common in Eastern Europe [9]. Mortality from these conditions has been rising in many European countries [8].

Like many other health-related behaviors, alcohol consumption is strongly patterned by socioeconomic status, as indicated by level of education, occupational class, and income. In many European countries, the frequency of drinking and sometimes also the levels of consumption are higher in higher socioeconomic groups, whereas problematic forms of consumption, like binge drinking, and alcohol-related health and social problems, are more frequent in lower socioeconomic groups [7,10].

Recent literature reviews have shown that mortality from alcohol-related conditions is higher in lower socioeconomic groups in most of the available studies [11,12], including studies from Finland [13], Sweden [14,15], the United Kingdom [16,17], and Estonia [18]. Comparative studies are, however, lacking, and it is therefore unknown whether there are important differences between countries in the magnitude of socioeconomic inequalities in alcohol-related mortality. Investigating whether there are differences is important because such differences might provide useful insights into the explanation of these inequalities, and into the potential for reducing them.

Studies of trends in inequalities in alcohol-related mortality are even scarcer. Finland is the only country in which detailed studies have been conducted. Over the past decades, the absolute inequality in alcohol-related mortality has strongly increased in Finland [19], which has contributed importantly to a rising inequality in total mortality and life expectancy [20,21]. These changes have been partly fueled by changes in the affordability of alcohol [22,23]. In Finland, the rise of inequality in alcohol-related mortality has partly undone the effect of declining inequality in smoking-related mortality, suggesting that one risk factor has been replaced by another [21]. Studies from other countries are very scarce [18,24], and it is therefore unknown whether the Finnish experience can be generalized to other countries.

To fill these gaps, we set out to study whether European countries differ in the magnitude of socioeconomic inequality in alcohol-related mortality, and what the trends of this inequality over the past decades have been. More specifically, we studied inequality in alcohol-related mortality by education level and occupational class in all European countries with available data, and assessed whether its magnitude and contribution to inequality in total mortality vary between countries and have changed over time.

## Methods

### Data

We extracted data from national or regional mortality registers in which deaths and population numbers were classified by socioeconomic position, such as the national longitudinal mortality registers in Finland, Sweden, Norway, and Denmark; the Office for National Statistics Longitudinal Mortality Study in England and Wales, and the Echantillon Démographique Permanent created by the National Institute of Statistics and Economic Studies (INSEE) in France. We included all European countries for which data on cause-specific mortality covering complete national or regional populations were available that allowed a distinction by the most commonly used indicator of socioeconomic position, level of education (see below). Unfortunately, not all European countries have data collection systems in place from which such data could be extracted (e.g., because of privacy restrictions or lack of information on education level in their population census), but our study includes 17 countries that together represent all parts of the European subcontinent. Table 1 gives an overview of the main characteristics of our data sources; S1–S3 Tables provide additional detail. To facilitate inspection of tables and graphs, we have grouped populations into four geographical regions (Northern Europe, Western Europe, Southern Europe, and Eastern Europe).

**Table 1:** Overview of data sources.

Geographical Region	Population	Design^1^	Years Covered by the Analysis^2^					
			1980–1984	1985–1989	1990–1994	1995–1999	2000–2004	2005–2009
**Northern Europe**	Finland	Longitudinal	1980–1985	1986–1990	1991–1995	1996–2000	2001–2005	2006–2010
	Sweden	Longitudinal			1990–1994	1995–1999	2000–2004	2005–2008
	Norway	Longitudinal	1980–1985	1985–1990	1990–1995	1995–2001	2001–2006	2006–2009
	Denmark	Longitudinal			1991–1995	1996–2000	2001–2005	
**Western Europe**	Scotland (UK)	Longitudinal			1991–1995	1996–2000	2001–2005	2006–2010
	England and Wales (UK)	Longitudinal	1981–1986	1986–1991	1991–1996	1996–2001	2001–2006	2006–2009
	Belgium	Longitudinal					2004–2005	
	France	Longitudinal	1980–1985	1985–1990	1990–1995	1995–1999	1999–2004	2004–2007
	Switzerland	Longitudinal			1990–1995	1995–2000	2000–2005	2005–2008
	Austria	Longitudinal	1981–1982		1991–1992		2001–2002	
**Southern Europe**	Barcelona (Spain)	Cross-sectional			1992–1996	1997–2001	2002–2006	2007–2010
	Basque Country (Spain)	Longitudinal				1996–2001	2001–2006	
	Madrid (Spain)	Longitudinal				1996–1997	2001–2003	
	Turin (Italy)	Longitudinal	1981–1986	1986–1991	1991–1996	1996–2001	2001–2006	2006–2010
**Eastern Europe**	Slovenia	Longitudinal			1991–1996		2002–2006	
	Hungary	Cross-sectional	1978–1981		1988–1991		1999–2002	
	Czech Republic	Cross-sectional					1998–2003	
	Poland	Cross-sectional					2001–2003	
	Lithuania	Cross-sectional, longitudinal^3^		1988–1990			2001–2005	2006–2009
	Estonia	Cross-sectional		1987–1991			1998–2002	

All data cover complete national or regional populations with the exceptions of Scotland (5.3% representative sample), England and Wales (1% representative sample), France (1% sample of individuals born in and living in mainland France), and Switzerland (Swiss nationals only). Age range is 35–79 y in all populations, with the exceptions of Norway (40–79 y), Poland (35–64 y), and Lithuania (35–69 y).

^1^Longitudinal design: population census linked to mortality during a follow-up period. Cross-sectional design: education level of deceased individuals from death certificates; person-years by education from population census.

^2^For each population, the periods covered by the data are given; because mortality follow-up periods often started and ended within a calendar year, the years given are approximate only. For the analysis, these periods have been allocated to the harmonized intervals given at the top of the columns.

^3^Lithuania had a cross-sectional design in 1988–1990 and 2000–2002 and a longitudinal design in 2001–2005 and 2006–2009. To remove design effects, longitudinal data were used for 2001–2005, and 1988–1990 data were adjusted using the differences between the two designs seen in 2000–2002 and 2001–2005.

Most data stemmed from longitudinal mortality follow-up after censuses in which socioeconomic information of the population at risk and of the deceased had been recorded; some countries had only unlinked cross-sectional data in which socioeconomic information on the population at risk came from the census, and socioeconomic information on the deceased came from the death certificate. In most countries linkage between the population and death registries was more than 95% complete; for Spain, where linkage failure exceeded 5%, mortality rates were multiplied by the inverse of the proportion of deaths that were successfully linked in order to adjust for the linkage failure. Data for Spain and Italy came from regional populations, but previous studies have shown that patterns observed in these regional populations correspond well to those seen at the national level [25–27]. The UK is represented by two regional populations: (1) England and Wales and (2) Scotland.

We used two indicators of socioeconomic position: level of education and occupational class. For education level, we used the International Standard Classification of Education 1997, and considered categories 0–2 (pre-primary, primary, and lower secondary education) as “low,” categories 3–4 (upper secondary and post-secondary non-tertiary education) as “middle,” and categories 5–6 (first and second stage of tertiary education) as “high” [28]. For occupational class, we used the Erikson-Goldthorpe-Portocarero scheme of occupational classes, and distinguished non-manual workers, manual workers, farmers, and self-employed workers [29]. Farmers and self-employed workers are heterogeneous groups in terms of their socioeconomic position (e.g., because some farmers and self-employed workers have employees working for them while others lead a precarious existence), which is also reflected in highly variable mortality rates in different European countries [30]. Therefore, we report on differences in mortality between manual and non-manual workers only. Because occupational class was not available for all populations, and information on occupational class is less reliable among women, we present results on mortality by occupational class (men only) in S5–S7 Tables rather than in the main analysis. Persons with missing education level or occupational class were excluded from the analysis. The proportion of the population aged 35–79 y with unknown education level varied between 0% and 11%; the percentage of the male population aged 35–64 y with unknown occupational class varied between 3% and 36% (S1–S3 Tables).

For our outcome variable, mortality due to alcohol-related causes, we selected four underlying causes of death: alcoholic psychosis, dependence, and abuse; alcoholic cardiomyopathy; alcoholic liver cirrhosis; and accidental poisoning by alcohol. In the most recent time period, most countries used ICD-10 (International Classification of Diseases–Tenth Revision) for classifying causes of death. ICD codes for alcohol-related conditions in ICD-10 and previous editions are given in S4 Table. All countries were able to provide data on all four alcohol-related causes in the most recent time period, but some were not able to for earlier periods in time.

### Analysis

Our analysis consisted of two parts: (1) a cross-population comparison of inequality in alcohol-related mortality for the most recent time period for which data were available in these populations and (2) a cross-population comparison of trends in the inequality in alcohol-related mortality over time. Mortality rates by education level and occupational class were age-standardized using the European Standard Population [31]. Because education level gradually loses its discriminatory power at higher ages, and because registration of causes of death becomes increasingly unreliable at higher ages, all analyses for education level are restricted to the age-range 35–79 y (for exceptions, see Table 1). Because registration of (former) occupation was incomplete for retired individuals, analyses for occupational class were restricted to the age range 35–64 y. All analyses were stratified by sex, because alcohol consumption patterns and inequality in these patterns are known to differ between men and women [32].

We systematically studied the magnitude of both relative and absolute inequalities because both are important [33,34], and we operationalized inequalities in terms of both a ratio and a difference measure of the excess mortality in lower as compared to higher socioeconomic groups. All measures were adjusted for age. For education level, we used the relative index of inequality (RII) and the slope index of inequality (SII) [35,36]. Both measures take into account all education groups (i.e., not just the lowest and highest groups) and derive from a linear regression analysis of mortality on education level. The RII was calculated using Poisson regression with the observed number of deaths as the dependent variable, person-years of population as the offset variable, and educational rank and dummy variables for age (in 5-y age groups) as independent variables. Educational rank was calculated for each education level (by population, sex, and time period) as the mean proportion of the population having a higher level of education. The RII is the ratio between mortality at rank 1 (the lowest education level) and rank 0 (the highest education level), and can thus be interpreted as the rate ratio of mortality between those with the very lowest and those with the very highest educational position in the population [35]. The SII represents the absolute version of the RII and was calculated as follows:

$$\text{SII}\;=\;\frac{2\times \text{ASMR}\times \left(\text{RII}-1\right)}{\left(\text{RII}+1\right)}$$ (1)

in which ASMR is the age-standardized mortality in the whole population [37]. The SII can be interpreted as the rate difference (in deaths per 100,000 person-years) between those with the very lowest and those with the very highest educational position in the population. The RII and SII take into account the size of education groups, and adjust the relative position of each group to its share in the population, which increases comparability over time and between populations if there are substantial differences in the distribution of the population across education groups [35]. The 95% CIs for RII and SII values were calculated using bootstrapping of 1,000 replicas [38]. The percent contribution of inequality in alcohol-related mortality to inequality in total mortality was calculated from the SIIs for alcohol-related mortality (numerator) and total mortality (denominator). As our comparison for occupational class involved only two classes (manual workers and non-manual workers), we used simple rate ratios and rate differences, with the non-manual worker class as the reference group for this socioeconomic indicator.

## Results

### Inequality in Alcohol-Related Mortality in the Most Recent Time Period

Levels of mortality from alcohol-related causes among men and women in the latest available time period are presented in Tables 2 and 3, respectively. Mortality from all alcohol-related causes together was highest in Hungary, both among men and women, with age-standardized mortality rates of 198.1 (95% CI 195.1–200.9) and 51.4 (95% CI 50.0–52.7) deaths per 100,000 person-years, respectively, mainly due to extremely high mortality from alcoholic liver cirrhosis. Alcoholic psychosis, dependence, and abuse mortality was highest in Denmark, and alcohol poisoning mortality was highest in Estonia.

**Table 2:** Latest observed mortality from alcohol-related causes among men aged 35–79 y, by population.

Geographical Region	Population (Time Period)	Age-Standardized Mortality Rate (95% CI)					Total Alcohol-Related Deaths as Percent of Total Mortality
		Alcoholic Cardiomyopathy	Alcoholic Liver Cirrhosis	Alcoholic Psychosis, Dependence, and Abuse	Alcohol Poisoning	Total Alcohol-Related Causes	
**Northern Europe**	Finland (2006–2010)	7.3 (6.7–7.9)	55.8 (54.0–57.5)	8.8 (8.1–9.5)	27.5 (26.2–28.8)	99.3 (96.9–101.6)	9.8%
	Sweden (2005–2008)	1.2 (1.0–1.4)	10.7 (10.1–11.4)	9.4 (8.8–10.0)	3.6 (3.3–4.0)	24.9 (23.9–25.9)	3.2%
	Norway (2006–2009)	—	9.7 (8.5–10.9)	—	—	25.7 (23.7–27.8)	2.7%
	Denmark (2001–2005)	—	37.6 (36.3–39.0)	35.1 (33.8–36.7)	0.6 (0.4–0.8)	74.5 (72.5–76.6)	6.5%
**Western Europe**	Scotland (2006–2010)	—	—	—	—	44.4 (37.5–51.8)	4.5%
	England and Wales (2006–2009)	0.7 (0.0–1.6)	15.2 (11.8–18.5)	1.9 (0.8–3.3)	1.1 (0.2–2.3)	18.9 (15.1–23.0)	2.3%
	Belgium (2004–2005)	—	19.5 (18.3–20.5)	7.4 (6.7–8.1)	—	28.0 (26.6–29.4)	2.5%
	France (2003–2007)	—	—	—	—	39.1 (33.5–44.8)	4.2%
	Switzerland (2005–2008)	1.0 (0.7–1.3)	16.8 (15.6–18.0)	6.6 (5.8–7.4)	0.4 (0.2–0.6)	24.8 (23.3–26.3)	3.3%
	Austria (2001–2002)	0.6 (0.3–0.9)	9.5 (8.2–10.9)	11.8 (10.3–13.2)	0.0 (0.0–0.2)	21.9 (19.8–24.0)	2.1%
**Southern Europe**	Barcelona (2007–2010)	0.4 (0.1–0.7)	6.8 (5.6–8.1)	0.8 (0.4–1.3)	0.2 (0.0–0.4)	8.1 (6.8–9.5)	1.0%
	Basque Country (2001–2006)	0.5 (0.2–0.8)	6.4 (5.5–7.4)	1.1 (0.7–1.5)	0.2 (0.0–0.4)	8.2 (7.1–9.3)	0.9%
	Madrid (2001–2003)	0.1 (0.0–0.2)	5.1 (4.1–6.1)	1.3 (0.8–1.9)	0.0 (0.0–0.0)	6.4 (5.3–7.6)	0.6%
	Turin (2006–2010)	0.1 (0.0–0.4)	2.7 (1.7–3.8)	1.3 (0.5–2.2)	0.0 (0.0–0.0)	4.2 (2.8–5.6)	0.6%
**Eastern Europe**	Slovenia (2002–2006)	6.2 (5.2–7.3)	48.7 (45.9–51.6)	20.9 (19.0–22.7)	—	75.7 (72.3–79.1)	6.2%
	Hungary (1999–2002)	12.2 (11.5–12.9)	166.2 (163.4–168.8)	19.1 (18.3–20.0)	0.7 (0.5–0.9)	198.1 (195.1–200.9)	9.0%
	Czech Republic (1998–2003)	0.3 (0.2–0.4)	27.1 (26.4–27.8)	3.0 (2.8–3.3)	3.6 (3.3–3.9)	34.0 (33.3–34.9)	2.1%
	Poland (2001–2003)	0.8 (0.7–0.9)	10.8 (10.3–11.3)	13.9 (13.4–14.4)	11.5 (11.0–11.9)	37.0 (36.2–37.8)	3.6%
	Lithuania (2006–2009)	24.0 (22.0–25.7)	65.8 (62.7–68.9)	3.9 (3.2–4.6)	45.8 (43.3–48.3)	139.4 (135.2–143.8)	7.4%
	Estonia (1998–2002)	48.2 (44.9–52.1)	27.9 (25.3–30.7)	8.6 (7.1–10.1)	58.1 (54.1–61.7)	142.8 (136.8–148.8)	5.9%
**All—median (interquartile range)**		0.8 (0.4–6.8)	16.0 (9.6–35.2)	7.4 (1.9–11.8)	0.7 (0.2–7.5)	31.0 (21.1–74.8)	3.3%

Age-standardized mortality rate in deaths per 100,000 person-years. Due to confidentiality restrictions, some countries did not supply information on causes of death that involved very small numbers of deaths (indicated by dashes).

**Table 3:** Latest observed mortality from alcohol-related causes among women aged 35–79 y, by population.

Geographical Region	Population (Time Period)	Age-Standardized Mortality Rate (95% CI)					Total Alcohol-Related Deaths as Percent of Total Mortality
		Alcoholic Cardiomyopathy	Alcoholic Liver Cirrhosis	Alcoholic Psychosis, Dependence, and Abuse	Alcohol Poisoning	Total Alcohol-Related Causes	
**Northern Europe**	Finland (2006–2010)	0.6 (0.5–0.8)	16.8 (15.8–17.8)	1.7 (1.4–2.0)	7.2 (6.6–7.9)	26.4 (25.2–27.6)	5.6%
	Sweden (2005–2008)	0.2 (0.1–0.2)	7.9 (7.0–8.7)	2.0 (1.8–2.4)	1.1 (0.9–1.3)	7.2 (6.7–7.7)	1.5%
	Norway (2006–2009)	—	4.4 (3.6–5.0)	—	—	9.0 (7.9–10.2)	1.6%
	Denmark (2001–2005)	—	16.0 (15.1–17.0)	9.4 (8.7–10.2)	0.3 (0.2–0.4)	25.9 (24.7–27.1)	3.5%
**Western Europe**	Scotland (2006–2010)	—	—	—	—	16.7 (12.2–20.9)	2.5%
	England and Wales (2006–2009)	0.0 (0.0–0.0)	8.4 (6.0–11.1)	1.9 (0.9–3.3)	0.1 (0.0–0.4)	10.5 (7.6–13.5)	1.8%
	Belgium (2004–2005)	—	8.6 (7.8–9.3)	2.9 (2.5–3.3)	—	11.6 (10.4–12.3)	1.9%
	France (2003–2007)	—	—	—	—	10.8 (8.0–13.6)	2.5%
	Switzerland (2005–2008)	0.1 (0.0–0.2)	7.9 (7.0–8.7)	2.2 (1.8–2.6)	0.1 (0.0–0.2)	10.2 (9.3–11.2)	2.4%
	Austria (2001–2002)	0.2 (0.0–0.4)	2.5 (1.8–3.2)	2.5 (1.8–3.2)	0.0 (0.0–0.1)	5.2 (4.3–6.3)	0.9%
**Southern Europe**	Barcelona (2007–2010)	0.1 (0.0–0.2)	2.2 (1.6–2.9)	0.2 (0.0–0.3)	0.1 (0.0–0.2)	2.5 (1.8–3.2)	0.7%
	Basque Country (2001–2006)	0.1 (0.0–0.2)	1.2 (0.8–1.5)	0.3 (0.1–0.5)	0.3 (0.1–0.5)	1.8 (1.3–2.3)	0.5%
	Madrid (2001–2003)	0 (0.0–0.0)	1.0 (0.6–1.4)	0.2 (0.0–0.3)	0.0 (0.0–0.0)	1.1 (0.7–1.6)	0.2%
	Turin (2006–2010)	0 (0.0–0.0)	0.9 (0.3–1.7)	0.1 (0.0–0.3)	0.0 (0.0–0.0)	1.0 (0.4–1.8)	0.3%
**Eastern Europe**	Slovenia (2002–2006)	0.8 (0.5–1.1)	16.3 (14.6–17.9)	3.5 (2.9–4.3)	—	20.4 (18.6–22.1)	3.6%
	Hungary (1999–2002)	1.9 (1.6–2.1)	46.5 (45.2–47.7)	2.9 (2.6–3.2)	0.1 (0.1–0.2)	51.4 (50.0–52.7)	5.0%
	Czech Republic (1998–2003)	0.1 (0.0–0.1)	7.5 (7.0–7.9)	0.7 (0.5–0.8)	1.0 (0.8–1.1)	9.2 (8.7–9.6)	1.1%
	Poland (2001–2003)	0.1 (0.0–0.1)	2.0 (1.8–2.2)	1.3 (1.1–1.4)	1.3 (1.2–1.5)	4.6 (4.3–4.9)	1.2%
	Lithuania (2006–2009)	5.9 (5.2–6.8)	24.2 (22.5–26.0)	0.7 (0.4–1.0)	12.1 (10.9–13.3)	42.9 (40.6–45.3)	6.9%
	Estonia (1998–2002)	13.3 (11.8–15.0)	12.0 (10.5–13.8)	2.2 (1.6–2.9)	14.6 (12.8–16.5)	42.1 (39.2–45.0)	4.3%
**All—median (interquartile range)**		0.1 (0.1–0.7)	7.9 (2.3–15.0)	1.9 (0.7–2.5)	0.3 (0.1–1.2)	10.3 (5.0–21.8)	1.9%

Age-standardized mortality rate in deaths per 100,000 person-years. Due to confidentiality restrictions, some countries did not supply information on causes of death that involved very small numbers of deaths (indicated by dashes).

**Fig 1:**
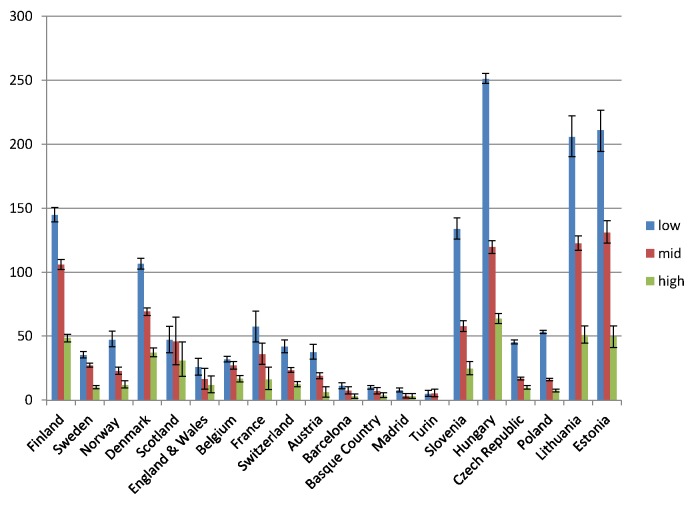
Latest observed mortality from alcohol-related causes among men aged 35–79 y, by population and level of education. The *y-*axis shows alcohol-related mortality, with 95% CIs, in deaths per 100,000 person-years.

**Fig 2:**
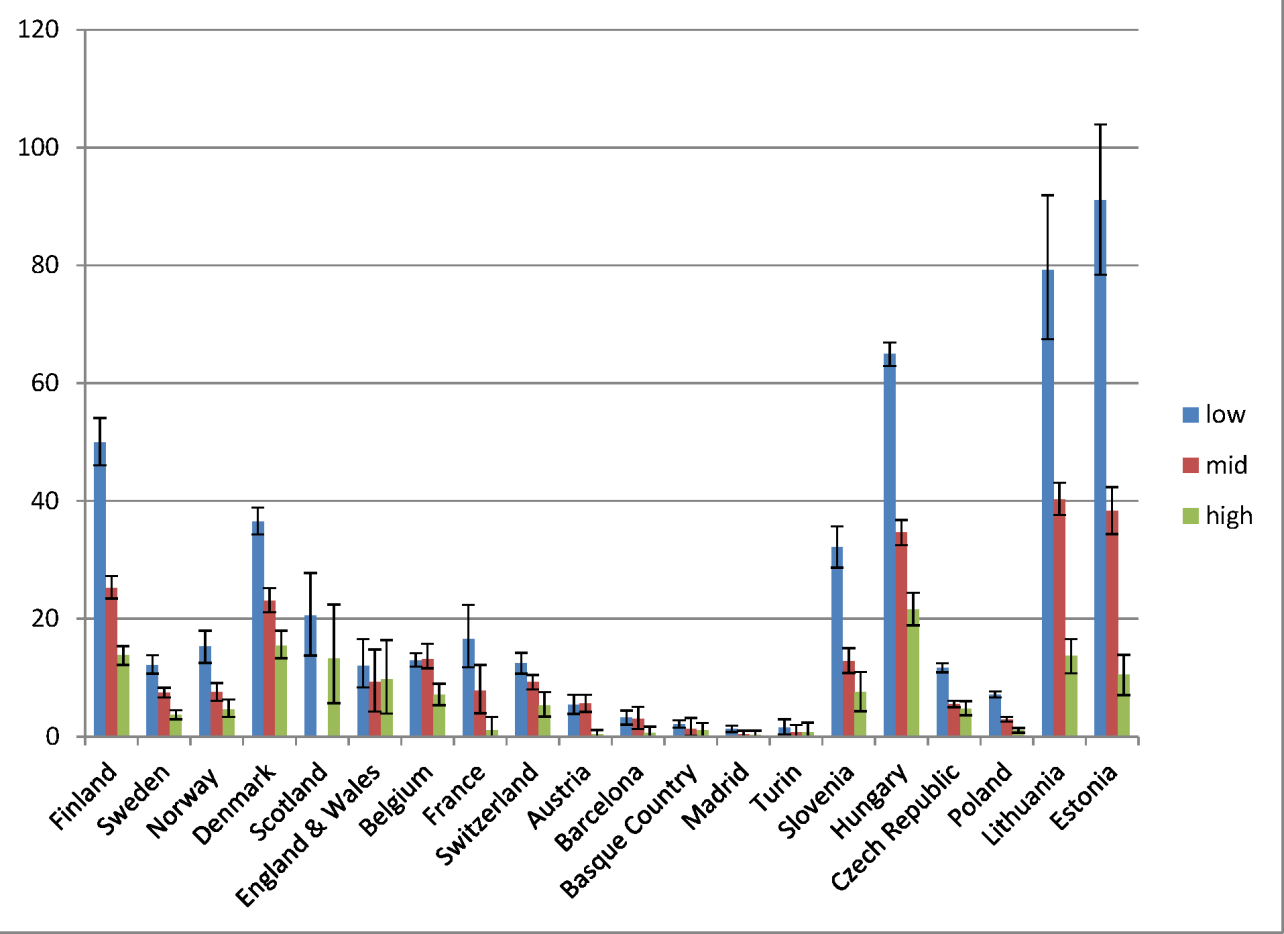
Latest observed mortality from alcohol-related causes among women aged 35–79 y, by population and level of education. The *y-*axis shows alcohol-related mortality, with 95% CIs, in deaths per 100,000 person-years. Among Scottish women in the middle education group, the number of deaths was smaller than ten, and because of confidentiality regulations, data could not be supplied for this group.

Educational inequality in mortality from alcohol-related causes was found in all populations, with mortality being higher among individuals in the middle education group than in the high education group, and among individuals in the low education group than in the middle education group, almost without exception (Figs 1 and 2). However, it can immediately be seen that the absolute gap in death rates between the low and the high education groups varied substantially between populations, being very small in the four populations in Southern Europe and very large in most countries in Eastern Europe. The gap in death rates between the low and high education groups was also relatively large in Finland and Denmark. Remarkably, alcohol-related mortality among individuals with a high level of education was also highly variable between populations.

Table 4 shows relative and absolute educational inequalities in mortality from alcohol-related causes in the most recent observed period. The RII almost always exceeded 1.00, indicating higher mortality among lower educated individuals. For example, among Finnish men the RII was 3.6 (95% CI 3.3–4.0) and the SII was 112.5 (95% CI 106.2–118.8) deaths per 100,000 person-years. As can be seen in Figs 1 and 2, the absolute inequality as measured by the SII was largest in Hungary, both among men and among women, with the two Baltic countries coming second and third. The absolute inequality in alcohol-related mortality was also very large in Slovenia, Finland, and Denmark.

**Table 4:** Age-adjusted educational inequality in mortality from alcohol-related causes in the most recent observed period, by population and sex, for individuals aged 35–79 y.

Geographical Region	Population (Period)	Men			Women		
		RII (95% CI)	SII (95% CI)	Percent of SII in Total Mortality	RII (95% CI)	SII (95% CI)	Percent of SII in Total Mortality
**Northern Europe**	Finland (2006–2010)	3.6 (3.3, 4.0)	112.5 (106.2, 118.8)	12.8%	5.6 (4.7, 6.8)	36.9 (33.8, 40.1)	10.3%
** **	Sweden (2005–2008)	3.8 (3.2, 4.4)	28.8 (26.1, 31.6)	5.5%	4.3 (3.2, 5.8)	9.0 (7.5, 10.5)	2.6%
** **	Norway (2006–2009)	6.4 (4.7, 8.6)	37.4 (31.4, 41.7)	4.5%	4.7 (2.9, 7.7)	11.7 (9.4, 14.3)	2.3%
** **	Denmark (2001–2005)	3.9 (3.5, 4.4)	88.1 (82.0, 94.3)	10.8%	3.8 (3.1, 4.6)	30.2 (26.5, 33.7)	5.5%
**Western Europe**	Scotland (2006–2010)	1.0 (0.5, 2.2)	1.5 (−25.7, 30.9)	0.3%	1.6 (0.5, 6.0)	7.4 (−10.2, 24.0)	2.3%
** **	England and Wales (2006–2009)	3.4 (1.4, 8.4)	20.8 (6.7, 32.1)	3.2%	1.5 (0.5, 4.4)	4.2 (−5.4, 14.3)	1.1%
** **	Belgium (2004–2005)	2.4 (1.9, 3.0)	23.3 (18.8, 27.8)	2.7%	1.9 (1.4, 2.7)	7.4 (3.8, 11.0)	1.9%
** **	France (2003–2007)	4.1 (2.3, 7.4)	47.7 (29.3, 61.4)	6.6%	10.3 (2.9, 36.5)	18.0 (10.7, 24.0)	6.0%
** **	Switzerland (2005–2008)	4.7 (3.7, 6.1)	32.3 (28.1, 36.1)	5.0%	2.3 (1.6, 3.3)	7.9 (4.4, 10.8)	3.5%
** **	Austria (2001–2002)	5.8 (3.9, 8.5)	30.8 (25.6, 35.4)	4.7%	1.4 (0.7, 3.1)	1.8 (−1.8, 5.3)	0.7%
**Southern Europe**	Barcelona (2007–2010)	6.0 (2.8, 13.2)	11.6 (8.0, 14.9)	1.9%	5.6 (1.3, 24.2)	3.4 (1.0, 5.1)	1.8%
** **	Basque Country (2001–2006)	3.7 (1.9, 7.2)	9.4 (5.3, 13.2)	1.9%	3.7 (0.7, 18.4)	2.1 (−0.3, 3.6)	1.5%
** **	Madrid (2001–2003)	5.9 (2.4, 14.4)	9.2 (5.7, 12.0)	1.5%	5.4 (0.5, 53.3)	1.5 (0.1, 2.7)	1.2%
** **	Turin (2006–2010)	2.5 (0.6, 10.1)	3.6 (−0.8, 7.8)	0.7%	3.0 (0.2, 49.7)	1.0 (−1.7, 2.8)	0.6%
**Eastern Europe**	Slovenia (2002–2006)	8.0 (6.6, 9.7)	117.8 (109.9, 124.9)	10.5%	8.5 (5.7, 12.7)	32.2 (28.0, 36.2)	8.2%
** **	Hungary (1999–2002)	7.2 (6.7, 7.8)	299.9 (292.6, 307.4)	10.9%	4.7 (4.2, 5.4)	67.0 (62.9, 70.8)	7.0%
** **	Czech Republic (1998–2003)	10.6 (9.0, 12.4)	56.2 (53.9, 58.5)	0.3%	4.8 (3.8, 6.2)	12.0 (10.4, 13.5)	0.2%
** **	Poland (2001–2003)	18.5 (16.2, 21.1)	66.4 (64.7, 68.3)	1.5%	10.1 (7.6, 13.5)	7.5 (6.9, 8.1)	0.6%
** **	Lithuania (2006–2009)	4.5 (3.9, 5.3)	177.6 (163.2, 191.0)	8.8%	6.9 (5.3, 9.0)	64.2 (58.0, 70.4)	9.1%
** **	Estonia (1998–2002)	4.5 (3.7, 5.4)	181.4 (162.8, 196.7)	5.1%	9.0 (6.6, 12.4)	67.4 (60.7, 73.9)	6.2%
**All—median (interquartile range)**		4.5 (3.7, 6.1)	34.8 (18.5, 94.2)	4.6%	4.7 (2.8, 6.0)	8.5 (4.0, 30.7)	2.3%

Table 4 also shows the contribution of alcohol-related mortality to inequalities in total mortality. That contribution was equal to or higher than 10% in Finland, Denmark (men only), Slovenia, and Hungary. Data on occupational class inequalities in alcohol-related mortality among men were available for 11 out of 17 countries, and show a pattern of consistently higher mortality among manual than non-manual workers. The contribution of alcohol-related mortality to occupational class inequalities in total mortality was often even larger than that seen for educational inequalities in total mortality, due to the restriction to a younger age group (S5 Table).

### Trends in Inequality in Alcohol-Related Mortality

Trends in inequality in alcohol-related mortality over time are illustrated in Tables 5–8. Relative inequality in alcohol-related mortality, as measured by the RII, has gone up in some populations, including Finland and Norway. The main change in inequality, however, is a strong rise in absolute inequality, as measured by the SII, in several countries in Eastern Europe (Hungary, Lithuania, and Estonia) and Northern Europe (Finland and Denmark). For example, among Finnish men the RII increased from 2.7 (95% CI 2.2–3.2) in 1980–1984 to 3.6 (95% CI 3.3–4.0) in 2005–2009, and over the same period the SII increased from 46.4 (95% CI 39.1–54.2) to 112.5 (95% CI 106.2–118.8). On the other hand, the absolute inequality in alcohol-related mortality has been stable in several other populations and has been declining among men in France.

**Table 5:** Trends in RII for educational inequality in mortality from alcohol-related causes among men aged 35–79 y, ca. 1980–2010, by population.

Geographical Region	Population	RII (95% CI)					
		1980–1984	1985–1989	1990–1994	1995–1999	2000–2004	2005–2009
**Northern Europe**	Finland	2.7 (2.2–3.2)	2.6 (2.2–3.0)	3.0 (2.6–3.5)	3.3 (2.9–3.7)	3.2 (2.9–3.5)	3.6 (3.3–4.0)
	Sweden			4.3 (3.6–5.2)	4.1 (3.5–4.8)	4.7 (4.0–5.4)	3.8 (3.2–4.4)
	Norway	2.6 (2.1–3.2)	2.9 (2.4–3.6)	3.9 (3.1–4.8)	4.2 (3.4–5.1)	5.2 (4.2–6.4)	6.4 (4.7–8.6)
	Denmark			3.0 (2.6–3.5)	2.7 (2.4–3.1)	3.9 (3.5–4.4)	
**Western Europe**	Scotland (UK)			N/E	3.1 (0.4–23.3)	3.2 (1.6–6.4)	1.0 (0.5–2.2)
	England and Wales (UK)					3.0 (1.3–6.9)	3.4 (1.4–8.4)
	France	8.8 (3.0–29.3)	5.7 (2.8–11.9)	6.9 (3.6–13.0)	9.2 (4.6–18.4)	4.2 (2.5–7.1)	4.1 (2.3–7.4)
	Switzerland			5.6 (4.7–6.7)	4.3 (3.6–5.1)	6.4 (5.3–7.8)	4.7 (3.7–6.1)
	Austria	4.7 (2.7–8.3)		5.4 (3.5–8.4)		5.8 (3.9–8.5)	
**Southern Europe**	Spain (Barcelona)			8.7 (3.7–20.5)	9.9 (4.4–22.1)	8.8 (4.1–18.6)	6.0 (2.8–13.2)
	Spain (Basque Country)				7.8 (3.4–18.2)	3.7 (1.9–7.2)	
	Spain (Madrid)				6.0 (2.0–18.1)	5.9 (2.4–14.4)	
	Italy (Turin)	11.2 (1.9–66.1)	2.0 (0.6–7.1)	5.0 (1.1–23.4)	5.2 (1.3–21.5)	7.3 (2.2–23.6)	2.5 (0.6–10.1)
**Eastern Europe**	Slovenia			14.1 (10.0–19.7)		8.0 (6.6–9.7)	
	Hungary	7.4 (6.0–9.1)		8.5 (7.6–9.5)		7.2 (6.7–7.8)	
	Lithuania		3.3 (2.2–4.9)			3.2 (2.7–3.9)	4.2 (3.5–5.0)
	Estonia		4.4 (3.1–6.4)			4.1 (3.2–5.1)	

Trend data for England and Wales are available only for the 2000s, because only two levels of education were available for the 1980s and 1990s. Trend data for Belgium, Czech Republic, and Poland are not available because data on alcoholic liver cirrhosis were missing in earlier periods. In Lithuania and Estonia, only mortality due to alcoholic liver cirrhosis and alcohol poisoning were included in this trend table, because comparable data on other alcohol-related causes were not available for earlier time periods. N/E, not estimable.

**Table 6:** Trends in SII for educational inequality in mortality from alcohol-related causes among men aged 35–79 y, ca. 1980–2010, by population.

Geographical Region	Population	SII (95% CI)					
		1980–1984	1985–1989	1990–1994	1995–1999	2000–2004	2005–2009
**Northern Europe**	Finland	46.4 (39.1, 54.2)	57.4 (49.5, 65.3)	66.6 (60.5, 73.4)	83.2 (76.9, 89.7)	87.1 (80.7, 94.0)	112.5 (106.2, 118.8)
** **	Sweden			33.4 (30.3, 36.5)	30.8 (28.2, 33.5)	33.5 (30.8, 35.8)	28.8 (26.1, 31.6)
** **	Norway	32.5 (25.0, 38.0)	42.3 (35.8, 48.7)	45.1 (39.0, 51.0)	43.8 (39.3, 48.4)	39.7 (35.7, 42.9)	37.4 (31.4, 41.7)
** **	Denmark			52.0 (45.6, 57.8)	56.0 (50.1, 61.8)	88.1 (82.0, 94.3)	
**Western Europe**	Scotland (UK)			18.6 (12.1, 26.5)	18.7 (−9.2, 38.9)	76.4 (34.4, 112.1)	1.5 (−25.7, 30.9)
** **	England and Wales (UK)					18.1 (3.2, 28.5)	20.8 (6.7, 32.1)
** **	France	94.1 (63.7, 117.2)	85.5 (59.3, 107.7)	66.4 (50.5, 79.5)	70.2 (54.9, 84.1)	53.4 (38.9, 66.5)	47.7 (29.3, 61.4)
** **	Switzerland			43.3 (39.6, 46.6)	39.4 (35.5, 43.1)	43.3 (40.1, 46.3)	32.3 (28.1, 36.1)
** **	Austria	17.6 (12.4, 21.8)		27.1 (21.5, 32.4)		30.8 (25.6, 35.4)	
**Southern Europe**	Spain (Barcelona)			13.4 (9.7, 16.3)	15.3 (11.5, 18.4)	14.1 (10.8, 17.0)	11.6 (8.0, 14.9)
** **	Spain (Basque country)				12.7 (9.1, 15.5)	9.4 (5.3, 13.2)	
** **	Spain (Madrid)				8.5 (4.8, 11.5)	9.2 (5.7, 12.0)	
** **	Italy (Turin)	10.8 (5.7, 14.3)	3.8 (−2.7, 9.5)	5.7 (1.3, 9.0)	6.8 (1.9, 10.6)	9.5 (5.0, 13.0)	3.6 (−0.8, 7.8)
**Eastern Europe**	Slovenia			150.0 (139.9, 159.2)		117.8 (109.9, 124.9)	
** **	Hungary	79 (74.2, 84.0)		235.2 (227.5, 242.2)		299.9 (292.6, 307.4)	
** **	Lithuania		26.8 (21.8, 31.7)			90.4 (79.0, 101.3)	137.7 (125.1, 148.2)
** **	Estonia		45.5 (36.9, 54.3)			104.1 (89.6, 116.9)	

Trend data for England and Wales are available only for the 2000s, because only two levels of education were available for the 1980s and 1990s. Trend data for Belgium, Czech Republic, and Poland are not available because data on alcoholic liver cirrhosis were missing in earlier periods. In Lithuania and Estonia, only mortality due to alcoholic liver cirrhosis and alcohol poisoning were included in this trend table, because comparable data on other alcohol-related causes were not available for earlier time periods.

**Table 7:** Trends in RII for educational inequality in mortality from alcohol-related causes among women aged 55–79 y, ca. 1980–2010, by population.

Geographical Region	Population	RII (95% CI)					
		1980–1984	1985–1989	1990–1994	1995–1999	2000–2004	2005–2009
**Northern Europe**	Finland	1.7 (1.1–2.7)	2.5 (1.8–3.6)	3.6 (2.7–4.9)	3.8 (2.9–4.8)	4.7 (3.8–5.8)	5.6 (4.7–6.8)
** **	Sweden			3.8 (2.6–5.6)	3.9 (2.8–5.3)	3.8 (2.9–5.0)	4.3 (3.2–5.8)
** **	Norway	1.5 (1.0–2.3)	1.2 (0.8–1.8)	2.4 (1.6–3.6)	3.1 (2.1–4.6)	4.9 (3.3–7.4)	4.7 (2.9–7.7)
** **	Denmark			2.3 (1.7–2.9)	2.8 (2.3–3.6)	3.8 (3.1–4.6)	
**Western Europe**	Scotland (UK)			4.8 (0.1–310.0)	6.9 (0.1–423.0)	2.7 (0.9–8.1)	1.6 (0.5–6.0)
** **	England and Wales (UK)					1.8 (0.6–5.3)	1.5 (0.5–4.4)
** **	France	6.6 (1.2–44.8)	7.1 (1.6–31.6)	2.2 (0.7–6.5)	6.1 (1.9–19.8)	4.5 (1.8–11.4)	10.3 (2.9–36.5)
** **	Switzerland			2.1 (1.6–2.8)	1.6 (1.2–2.0)	1.7 (1.2–2.2)	2.3 (1.6–3.3)
** **	Austria	5.3 (1.3–22.2)		1.3 (0.5–3.7)		1.4 (0.7–3.1)	
**Southern Europe**	Spain (Barcelona)			4.8 (0.8–27.9)	21.8 (3.0–157.5)	7.9 (1.7–36.8)	5.6 (1.3–24.2)
** **	Spain (Basque country)				3.1 (0.6–16.0)	3.7 (0.7–18.4)	
** **	Spain (Madrid)				1.1 (0.2–7.8)	5.4 (0.5–53.3)	
** **	Italy (Turin)	4.4 (0.1–273.3)	0.5 (0.0–4.2)	3.0 (0.2–38.7)	61.0 (0.9–3,955.3)	3.0 (0.5–16.8)	3.0 (0.2–49.7)
**Eastern Europe**	Slovenia			10.0 (5.4–18.3)		8.5 (5.7–12.7)	
** **	Hungary	5.1 (3.3–8.1)		4.7 (3.7–5.3)		4.4 (4.2–5.4)	
** **	Lithuania		8.0 (3.5–18.3)			6.9 (5.1–9.3)	6.6 (4.9–8.7)
** **	Estonia		8.1 (4.0–16.8)			8.8 (5.9–13.2)	

Trend data for England and Wales are available only for the 2000s, because only two levels of education were available for the 1980s and 1990s. Trend data for Belgium, Czech Republic, and Poland are not available because data on alcoholic liver cirrhosis were missing in earlier periods. In Lithuania and Estonia, only mortality due to alcoholic liver cirrhosis and alcohol poisoning were included in this trend table, because comparable data on other alcohol-related causes were not available for earlier time periods.

**Table 8:** Trends in SII for educational inequality in mortality from alcohol-related causes among women aged 35–79 y, ca. 1980–2010, by population.

Geographical Region	Population	SII (95% CI)					
		1980–1984	1985–1989	1990–1994	1995–1999	2000–2004	2005–2009
**Northern Europe**	Finland	4.3 (1.0, 7.4)	10.4 (6.3, 13.9)	14.8 (11.8, 17.8)	20.4 (17.1, 23.6)	29.0 (26.0, 32.0)	36.9 (33.8, 40.1)
** **	Sweden			7.3 (4.6, 10.0)	4.8 (1.8, 7.5)	4.9 (1.7, 7.7)	7.9 (4.4, 10.8)
** **	Norway	3.7 (−0.0, 6.8)	2.1 (−1.9, 6.5)	8.0 (4.2, 11.1)	9.5 (6.5, 12.8)	11.2 (9.0, 12.9)	11.7 (9.4, 14.3)
** **	Denmark			15.0 (10.8, 19.1)	21.4 (17.3, 25.5)	30.2 (26.5, 33.7)	
**Western Europe**	Scotland (UK)			5.6 (−4.1, 11.8)	10.1 (−5.7, 18.2)	27.6 (−1.2, 50.4)	7.4 (−10.2, 24.0)
** **	England and Wales (UK)					4.9 (−4.0, 12.9)	4.2 (−5.4, 14.3)
** **	France	28.7 (5.8, 42.2)	30.7 (12.4, 42.6)	10.6 (−2.4, 22.5)	22.1 (10.9, 30.8)	19.1 (7.9, 27.2)	18.0 (10.7, 24.0)
** **	Switzerland			7.3 (4.6, 10.0)	4.8 (1.8, 7.5)	4.9 (1.7, 7.7)	7.9 (4.4, 10.8)
** **	Austria	3.7 (1.2, 5.6)		1.0 (−1.8, 4.0)		1.8 (−1.8, 5.3)	
**Southern Europe**	Spain (Barcelona)			2.7 (0.2, 4.3)	4.5 (2.8, 6.0)	3.4 (1.4, 4.9)	3.4 (1.0, 5.1)
** **	Spain (Basque country)				2.2 (−1.1, 4.4)	2.1 (−0.3, 3.6)	
** **	Spain (Madrid)				0.1 (−1.7, 2.3)	1.5 (0.1, 2.7)	
** **	Italy (Turin)	1.4 (−1.2, 3.1)	−1.2 (−3.2, 3.0)	1.5 (−1.5, 3.8)	3.4 (1.6, 5.0)	2.5 (−0.1, 5.0)	1.0 (−1.7, 2.8)
**Eastern Europe**	Slovenia			39.6 (33.5, 45.0)		32.2 (28.0, 36.2)	
** **	Hungary	17.4 (13.9, 20.3)		55.0 (50.2, 59.8)		67.0 (62.9, 70.8)	
** **	Lithuania		9.0 (6.6, 10.9)			40.4 (35.7, 44.7)	53.3 (47.3, 58.4)
** **	Estonia		12.6 (9.5, 15.6)			42.4 (36.4, 47.7)	

Trend data for England and Wales are available only for the 2000s, because only two levels of education were available for the 1980s and 1990s. Trend data for Belgium, Czech Republic, and Poland are not available because data on alcoholic liver cirrhosis were missing in earlier periods. In Lithuania and Estonia, only mortality due to alcoholic liver cirrhosis and alcohol poisoning were included in this trend table, because comparable data on other alcohol-related causes were not available for earlier time periods.

S6 and S7 Tables show trends in relative and absolute inequality in alcohol-related mortality by occupational class. Here again, we observe a strong rise of absolute inequality among men in several countries/regions, including Finland, England and Wales, and Lithuania.

**Fig 3:**
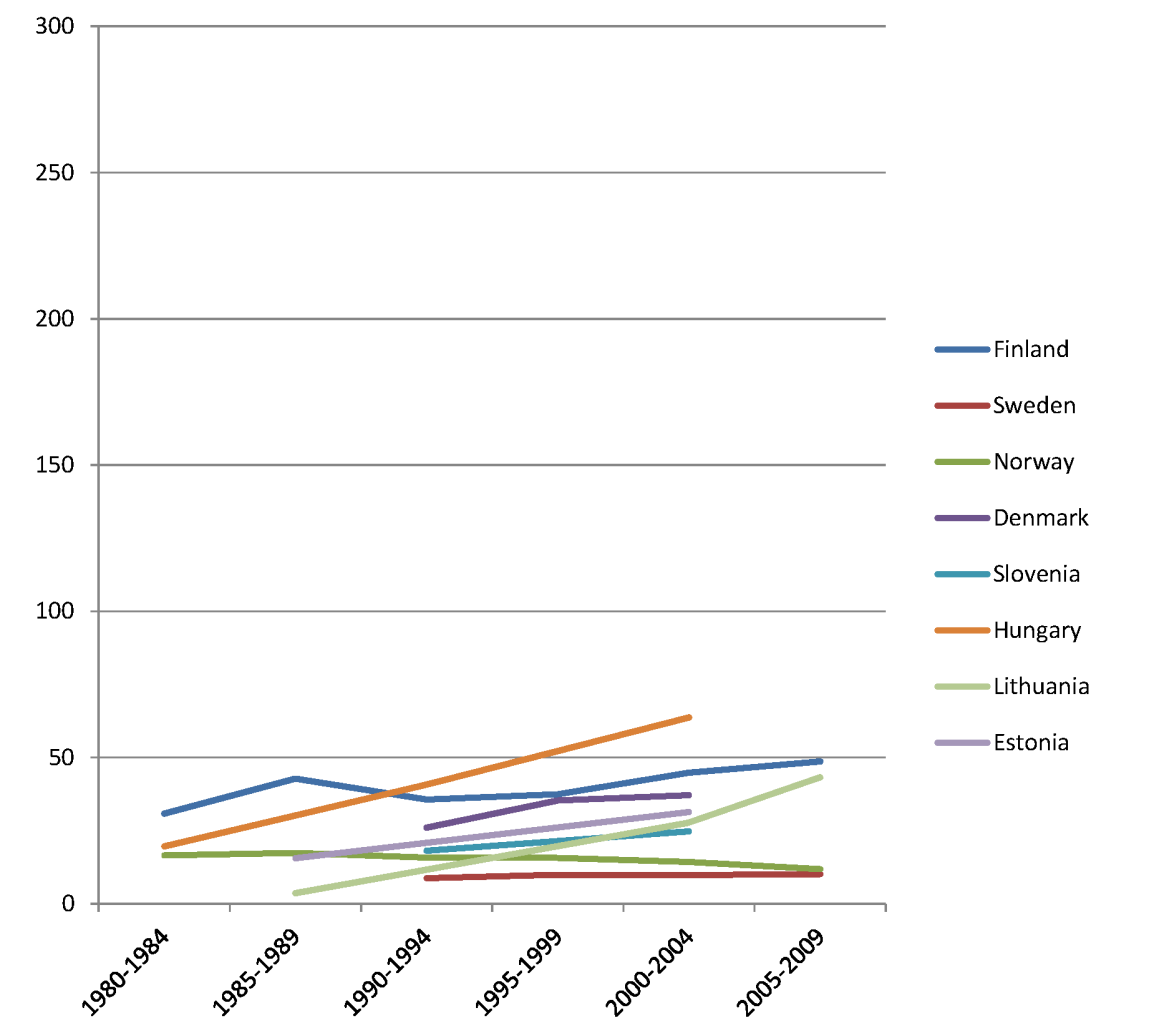
Trends in age-adjusted mortality from alcohol-related causes among men aged 35–79 y in the high education group in Northern and Eastern European countries, ca. 1980–2010, by country. The y-axis shows age-adjusted alcohol-related mortality in deaths per 100,000 person-years.

**Fig 4:**
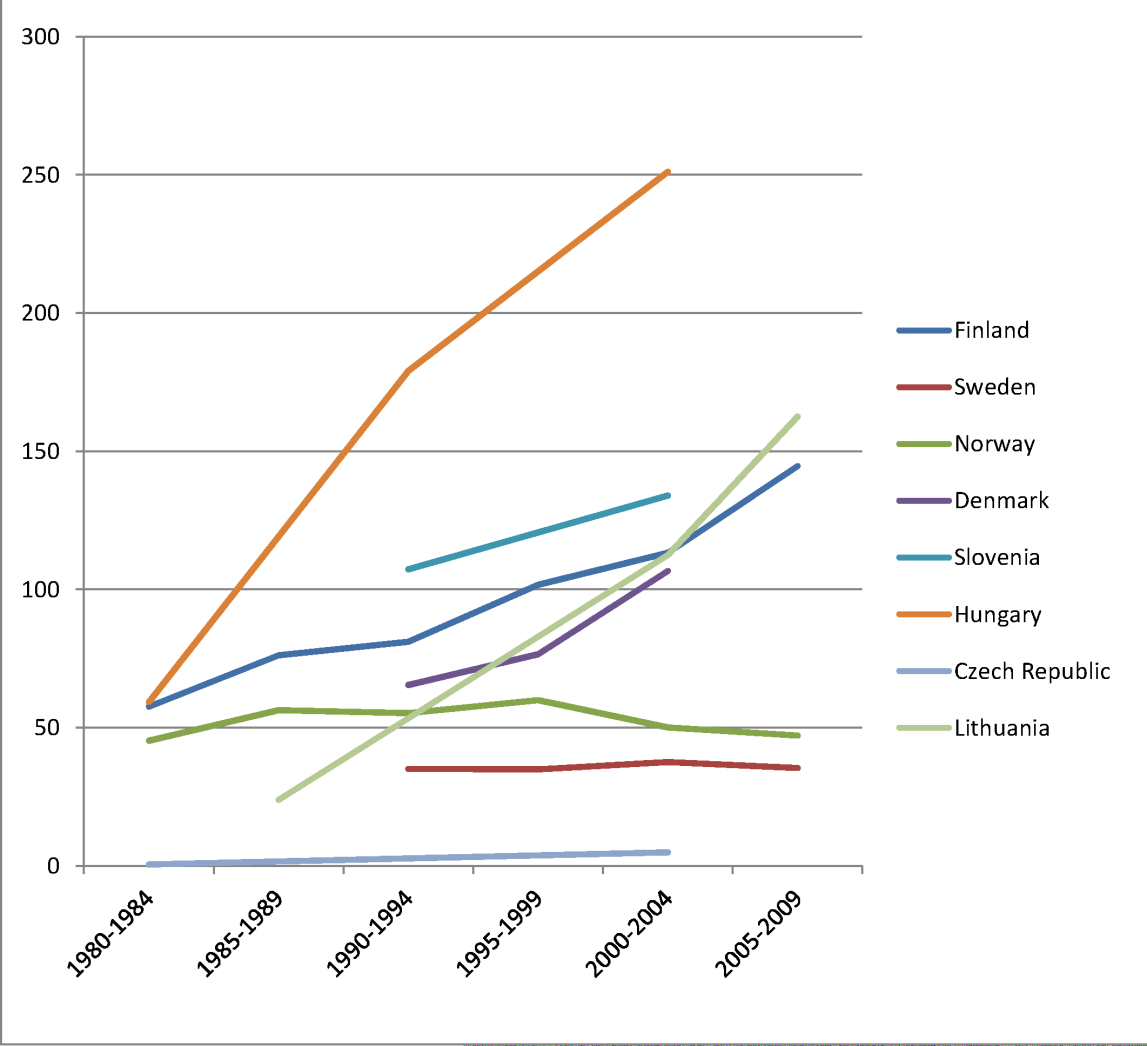
Trends in age-adjusted mortality from alcohol-related causes among men aged 35–79 y in the low education group in Northern and Eastern European countries, ca. 1980–2010, by country. The *y-*axis shows age-adjusted alcohol-related mortality in deaths per 100,000 person-years.

**Fig 5:**
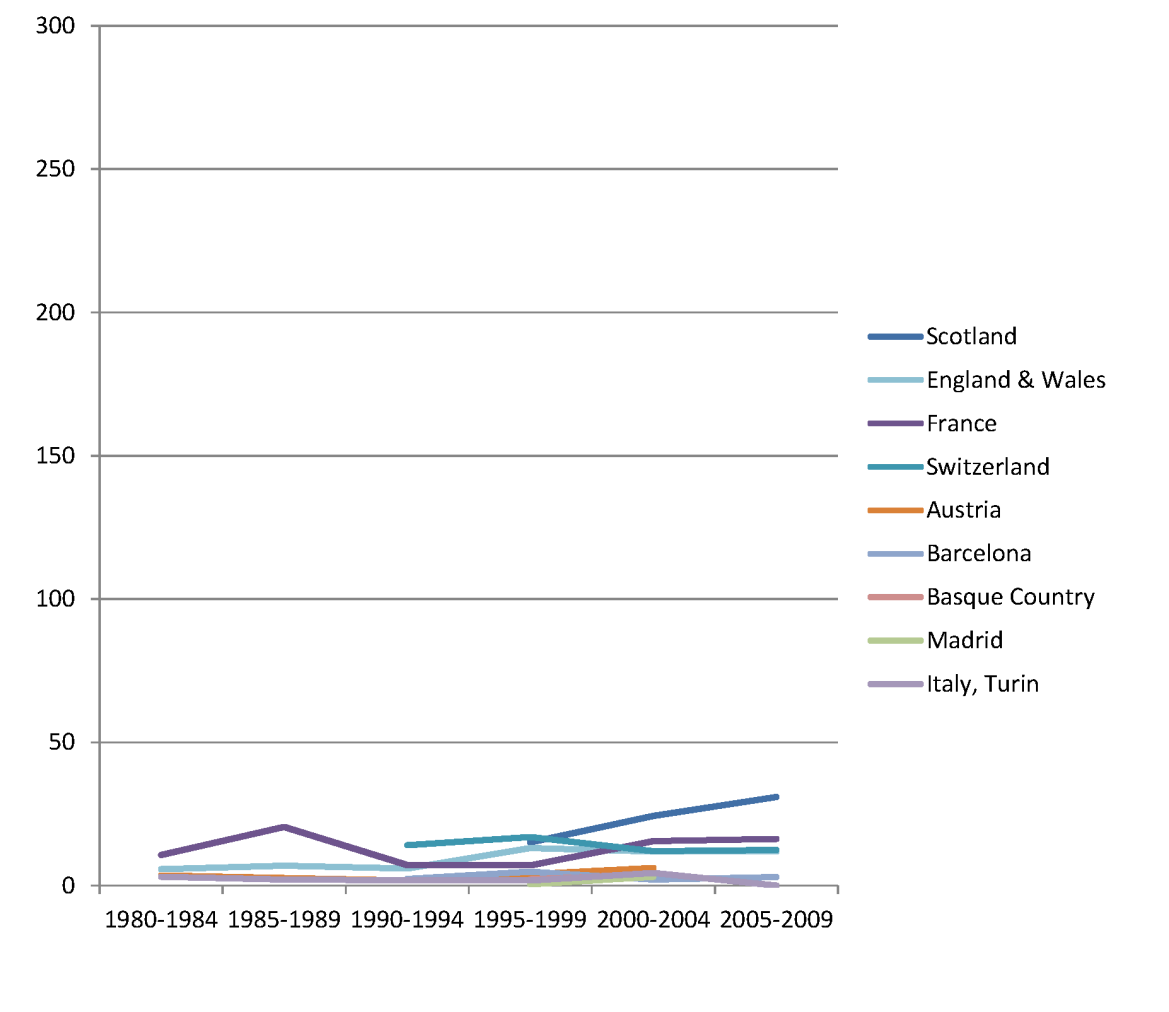
Trends in age-adjusted mortality from alcohol-related causes among men aged 35–79 y in the high education group in Western and Southern European countries/regions, ca. 1980–2010, by country/region. The *y-*axis shows age-adjusted alcohol-related mortality in deaths per 100,000 person-years.

**Fig 6:**
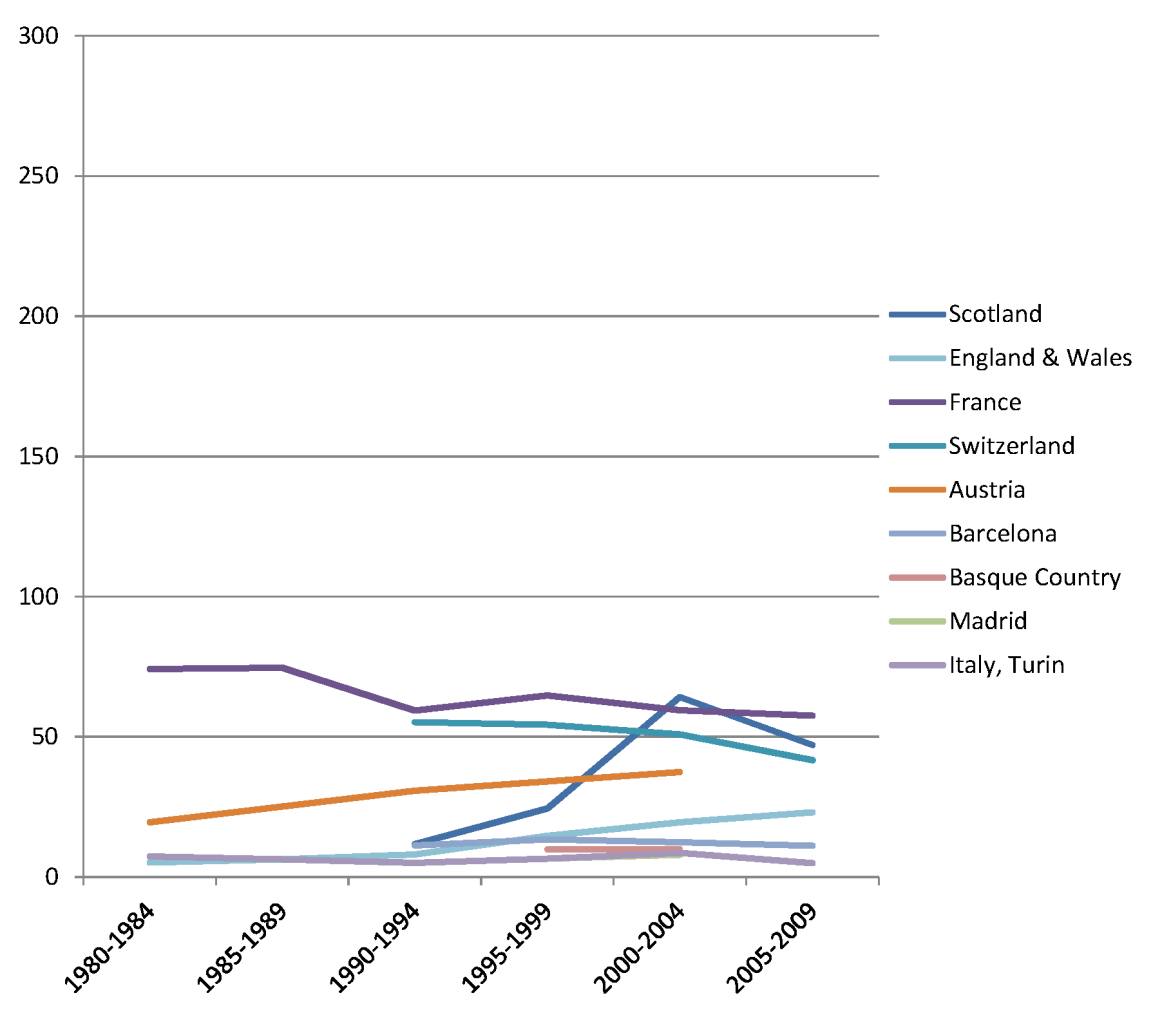
Trends in age-adjusted mortality from alcohol-related causes among men aged 35–79 y in the low education group in Western and Southern European countries/regions, ca. 1980–2010, by country/region. The *y-*axis shows age-adjusted alcohol-related mortality in deaths per 100,000 person-years. In England and Wales, individuals in the low and middle education groups could not be distinguished before 2000, and therefore both groups have been combined in the low education group in all study periods.

The rise of absolute inequality in alcohol-related mortality in several populations was due to a remarkable increase in mortality from alcohol-related conditions among lower educated individuals, with stable or only moderately increasing mortality among higher educated individuals, as shown in Figs 3–6. However, trends are far from uniform. For example, while mortality among men in the low education group has risen enormously in Finland and Denmark, it has been relatively stable in Sweden and Norway. The stability, or even tendency towards decline, of mortality from alcohol-related causes among lower educated men in France, Switzerland, Spain, and Italy is also noteworthy.

**Fig 7:**
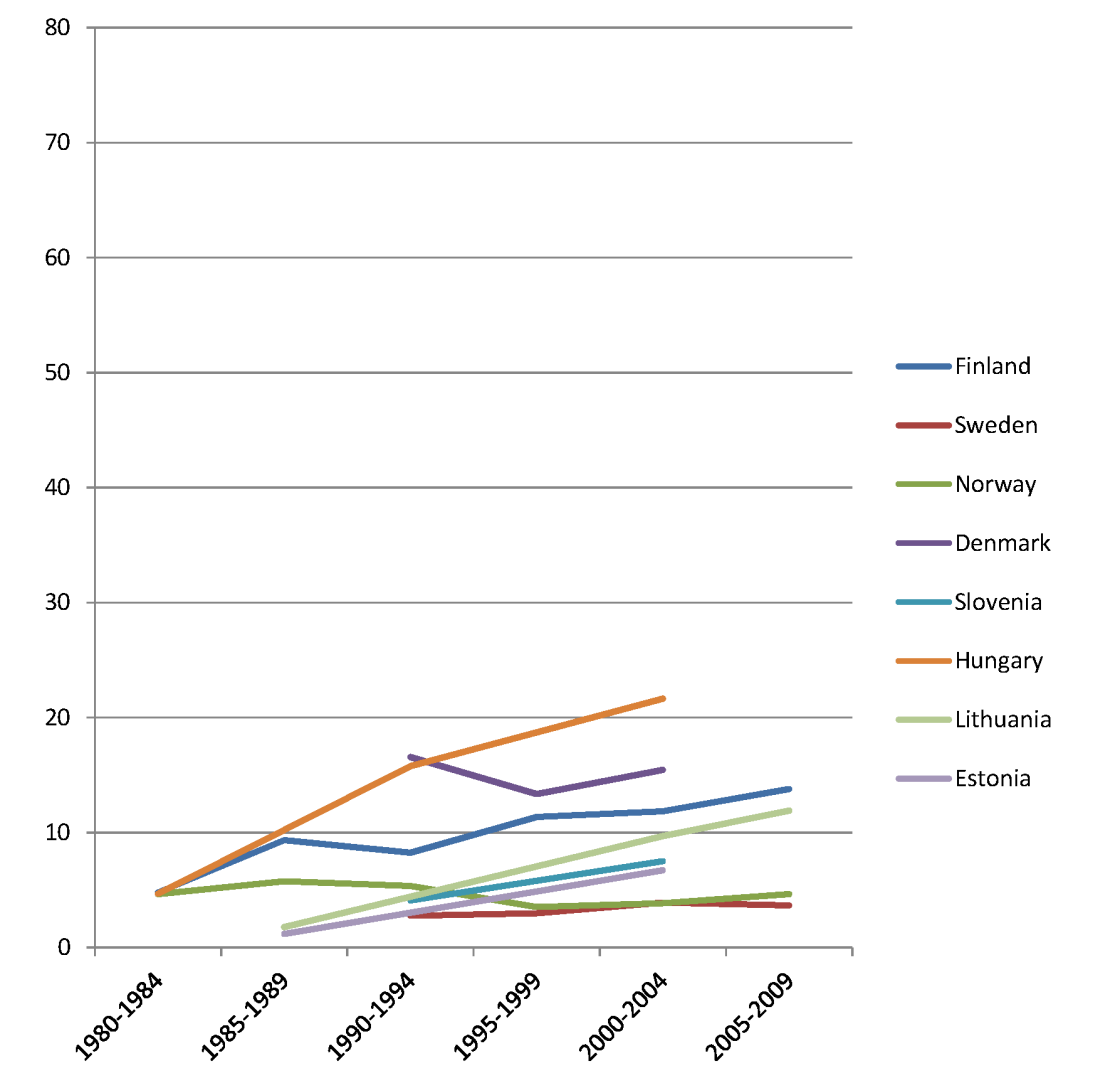
Trends in age-adjusted mortality from alcohol-related causes among women aged 35–79 y in the high education group in Northern and Eastern European countries, ca. 1980–2010, by country. The *y-*axis shows age-adjusted alcohol-related mortality in deaths per 100,000 person-years.

**Fig 8:**
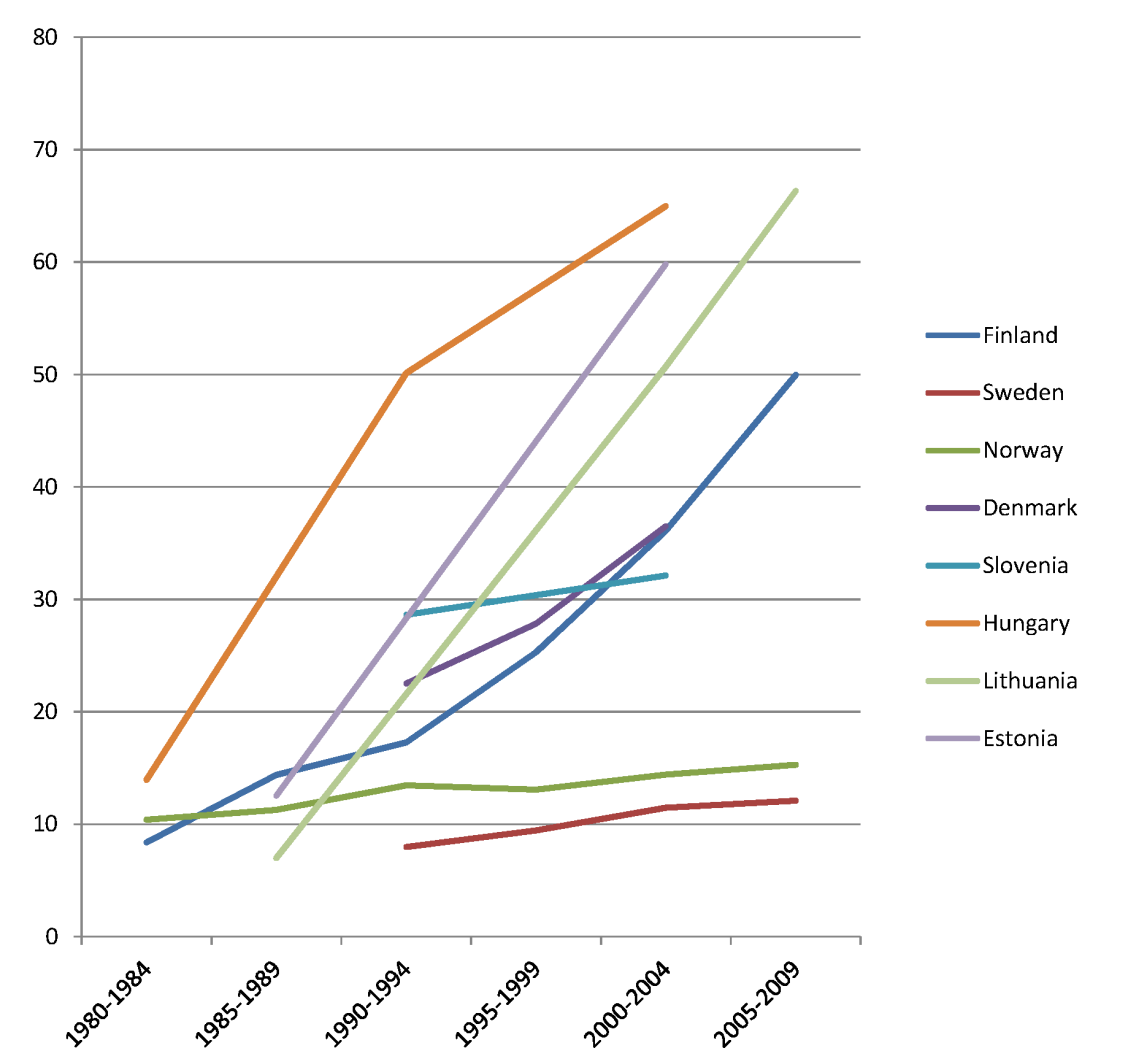
Trends in age-adjusted mortality from alcohol-related causes among women aged 35–79 y in the low education group in Northern and Eastern European countries, ca. 1980–2010, by country. The *y-*axis shows age-adjusted alcohol-related mortality in deaths per 100,000 person-years.

**Fig 9:**
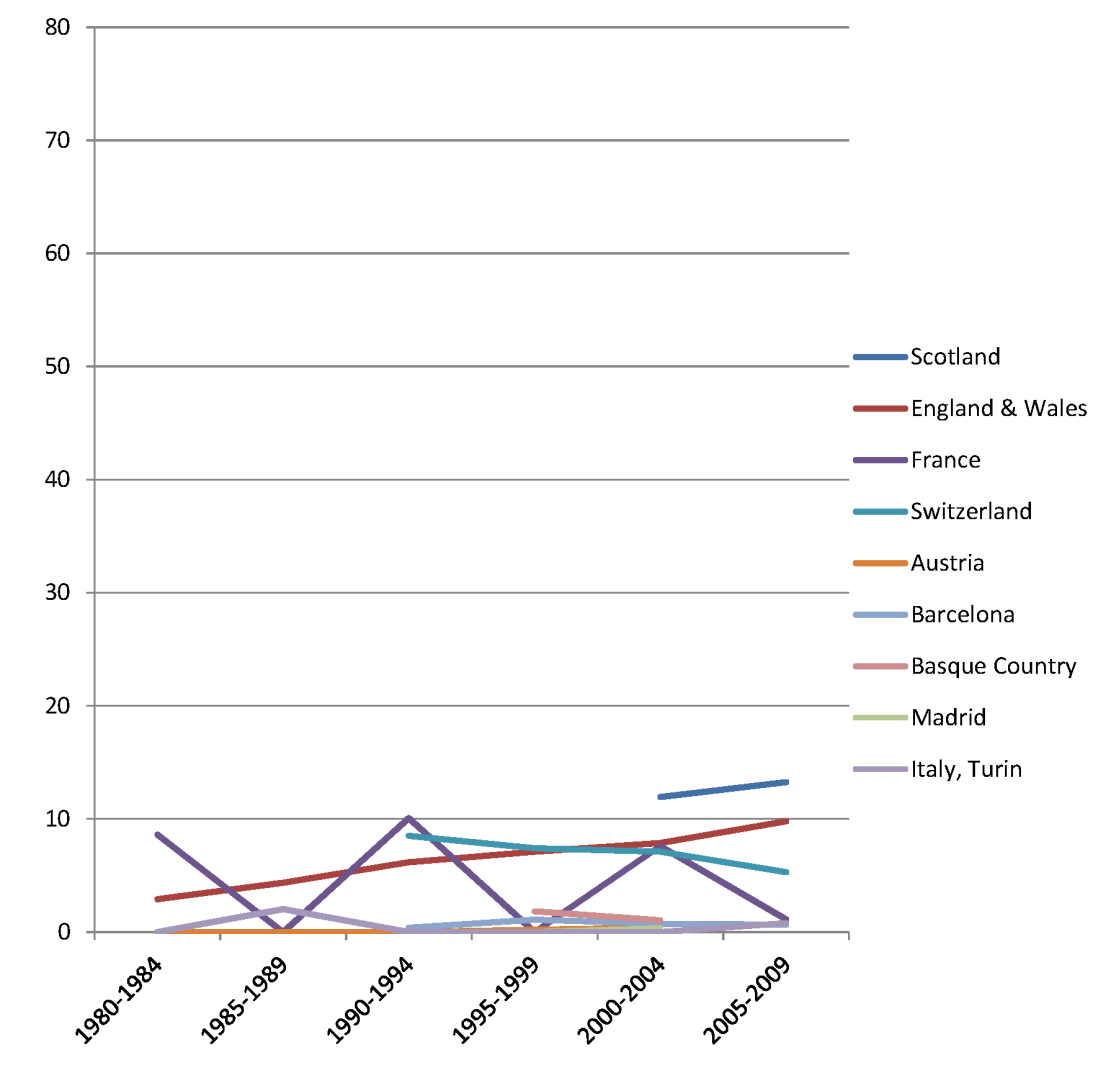
Trends in age-adjusted mortality from alcohol-related causes among women aged 35–79 y in the high education group in Western and Southern European countries/regions, ca. 1980–2010, by country/region. The *y-*axis shows age-adjusted alcohol-related mortality in deaths per 100,000 person-years.

**Fig 10:**
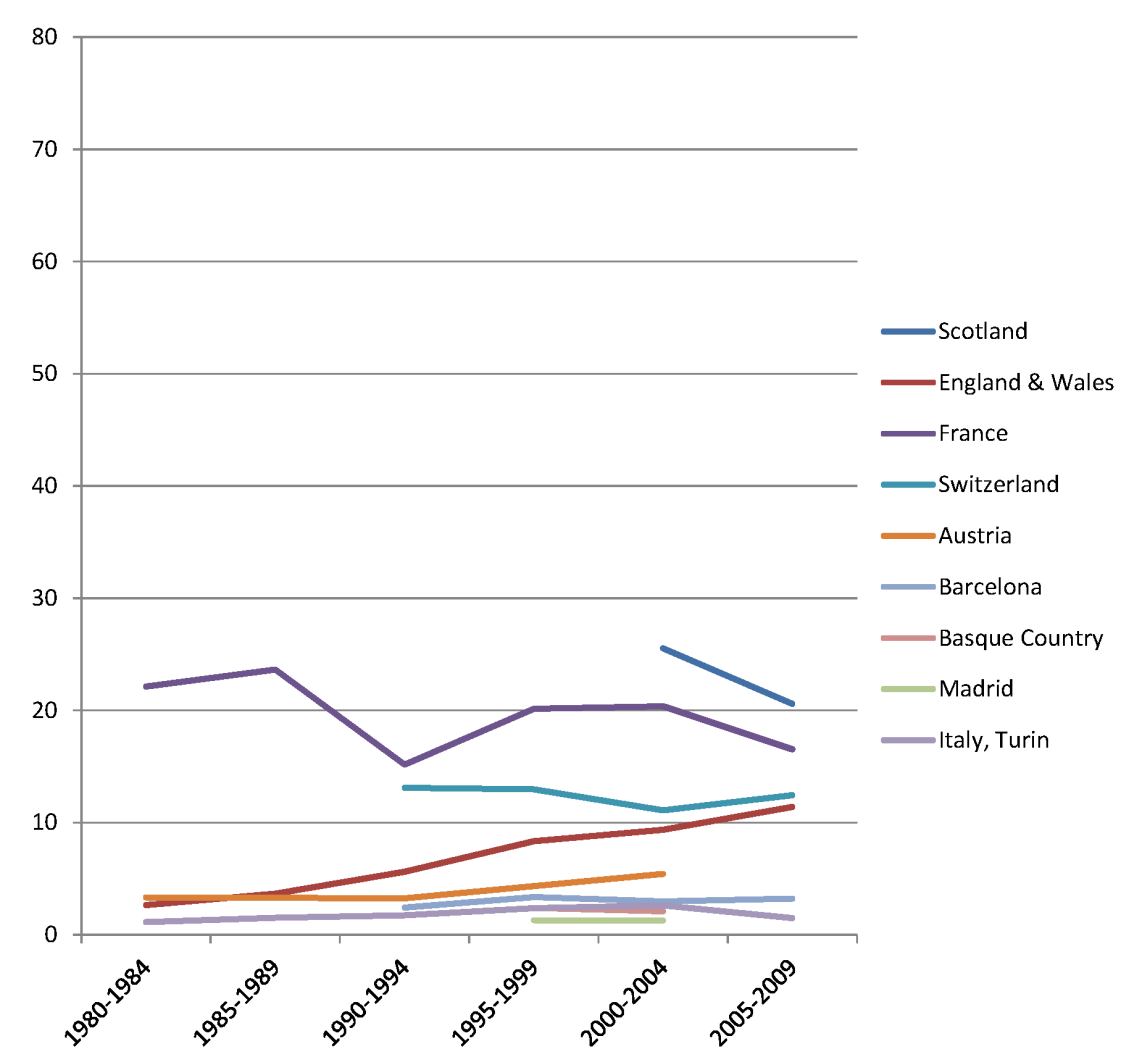
Trends in age-adjusted mortality from alcohol-related causes among women aged 35–79 y in the low education group in Western and Southern European countries/regions, ca. 1980–2010, by country/region. The *y-*axis shows age-adjusted alcohol-related mortality in deaths per 100,000 person-years. In England and Wales, individuals in the low and middle education groups could not be distinguished before 2000, and therefore both groups have been combined in the low education group in all study periods.

Similar results are seen for women (Figs 7–10) and for men by occupational class (S1 and S2 Figs).

## Discussion

### Summary of Main Findings

Rates of alcohol-related mortality are higher in lower socioeconomic groups (as measured by education level and occupational class) in all countries. Both relative and absolute inequalities are largest in Eastern Europe, and Finland and Denmark also have very large absolute inequalities in alcohol-related mortality. Over time, the relative inequality in alcohol-related mortality has increased in many countries, but the main change is a strong rise in the absolute inequality in several countries in Eastern Europe (Hungary, Lithuania, and Estonia) and Northern Europe (Finland and Denmark) due to a rapid rise in alcohol-related mortality in lower socioeconomic groups. In some of these countries alcohol-related causes now account for 10% or more of the inequality in total mortality in the age group 35–79 y.

### Strengths and Limitations

This study is unique in its population-wide coverage of a wide range of European countries and a long period of time, using two indicators of socioeconomic position: education level and occupational class. The main limitation is that we had to rely on routinely registered data, and that cause-of-death data are a far from perfect source of information on alcohol-related mortality.

It has been suggested that differences between European countries in the level of mortality from alcohol-related conditions are likely to be at least partly due to different recording practices [8]. Between-country differences in mortality levels as observed in the present study—which also strongly influence the absolute inequality in mortality from alcohol-related conditions—should therefore be regarded with caution. For example, the quality and coverage of alcohol-related cause-of-death data have been characterized as particularly good in Finland [19], which may partly be due to better recognition of alcohol-related conditions or a relative absence of social stigma associated with alcohol abuse in this country. By contrast, recording whether excessive alcohol consumption is the cause of liver cirrhosis may be far from complete in other countries [39,40].

Although under-recording is probably the most prevalent source of bias, over-recording has been reported in Hungary, where liver cirrhosis was often assigned as a cause of death in cases of diagnostic uncertainty [41]. To redress this problem, a change in coding rules was implemented in 1996. From our experience working with cause-of-death statistics in Hungary (K. K.), this resulted in a substantial drop in mortality from alcoholic liver cirrhosis. This implies that the mortality rate from alcohol-related causes in Hungary in the early 1980s and early 1990s, as presented in Tables 2 and 3 and Figs 3, 4, 7 and 8, was overestimated, and that the real increase was even larger than suggested by our data.

Over- and under-recording may also differ between socioeconomic groups or change over time. For example, the lower mortality rate from alcohol-related causes among those with higher socioeconomic position could be partly due to social stigma, and to medical doctors being more hesitant to attach social stigma to people of social standing similar to their own [13]. Yet, it is likely that the risk of bias is less for between-country comparisons of the relative inequality in alcohol-related mortality, and for within-country comparisons of relative and absolute inequality over time, than for between-country comparisons of the absolute inequality in alcohol-related mortality.

The second limitation with the cause-of-death data is that we have studied directly alcohol-attributable causes of death only, and these cover only a part of all alcohol-attributable deaths. The Global Burden of Disease study estimated that alcohol accounts for around 11% of deaths among men in European populations, many of which are from cardiovascular disease and cancer [42]. In our data the alcohol-related causes generally account for a few percentage points only, partly because we have not taken into account such more indirect effects (Tables 2 and 3). In Finnish studies, this problem has to some extent been reduced by using data on contributory causes of death [13,19], but in international comparisons these data are not available and, even if they were, may present even greater problems of comparability.

Another limitation is that although we used the most recent data available when we undertook this study (2012–2014), some of the data are already quite old, particularly in Eastern Europe. Also, whereas data on level of education were relatively complete, data on occupational class were more often missing, particularly in Lithuania (36% missing) and Estonia (35% missing) (S2 Table). Despite the greater risk of bias that this creates, the fact that occupational class data produce findings similar to those of educational data increases confidence in our findings.

### Interpretation

Excessive alcohol consumption is one of the greatest challenges for public health in Europe—in no other world region is the alcohol-attributable burden of disease higher than in Europe [43]. As our study shows, alcohol-related health problems have also become an important driver of socioeconomic inequality in mortality in many parts of the region, particularly in Eastern Europe and in the Nordic countries. Our previous study of cause-specific inequality in mortality in Europe in the 1990s already pointed in this direction [37], but our present study shows that this is also a rapidly growing problem.

The omnipresence of socioeconomic inequality in alcohol-related mortality in Europe suggests that it is linked to structural characteristics of societies, but its specific explanation is likely to be complex. First, it is important to recognize that part of the association may be due to selection factors: alcohol-related physical, mental, and social problems may lead to a lower socioeconomic position, although this is likely to affect income more than education level and occupational class [13]. Furthermore, certain personality traits or psychological problems in childhood and adolescence may predispose both to a higher risk of problematic drinking and to lower educational level [13,14].

It is highly plausible, however, that the association between low socioeconomic status and alcohol-related health problems also partly reflects an indirect causal effect of low socioeconomic position on the risk of dying from alcohol-related conditions, both through a higher prevalence of harmful consumption levels or patterns and through a higher vulnerability to the effects of alcohol. The first may be due to a higher exposure to material and psychosocial stressors, with alcohol being used for tension reduction [44,45]. It has also been suggested that individuals from lower socioeconomic groups, for example, in Hungary [46] and other Eastern European countries, are more exposed to lower quality alcoholic drinks, such as home-brewed and non-beverage alcohol, that may contain toxic compounds [47–49].

A higher vulnerability to the effects of alcohol in individuals from lower socioeconomic groups is suggested by a Finnish longitudinal study, which found that, with consumption level and drinking patterns controlled, alcohol-related hospitalizations and mortality were much more frequent in lower socioeconomic groups [50], perhaps because of an interaction with dietary habits, safety of the drinking environment, or support from work and family to address emerging alcohol problems.

We found that the inequality in alcohol-related mortality is much larger in some populations in Northern and Eastern Europe than it is in Western and Southern Europe. Part of the explanation for this may lie in different drinking cultures [51]. Binge drinking is common in Northern and Eastern Europe, and strongly socially stratified, whereas in Southern Europe, a Mediterranean style of drinking—characterized by almost daily drinking of alcohol, mostly in the form of wine with meals, and less acceptance of public drunkenness—still predominates, particularly in older generations, including lower socioeconomic groups [4,32]. Nevertheless, daily drinking of moderate amounts of alcohol does increase the risk of cirrhosis [52] and may be the main factor behind the inequality in alcohol-related mortality in Southern Europe, which is indeed dominated by cirrhosis mortality (Tables 2 and 3). We also found remarkable differences between men and women: whereas the relative inequality in alcohol-related mortality is sometimes larger among women than among men, the absolute inequality is almost always larger among men (Table 4).

Inequalities in alcohol-related mortality have been very dynamic, as shown by this study, which at the very least suggests that they are not immutable. To what extent do changes in the inequality in alcohol-related mortality reflect policy impacts? Trends in alcohol control policies in Western Europe were analyzed in the European Comparative Alcohol Study [53]. Between 1950 and 2000, alcohol control became less strict in Northern Europe—partly as a result of harmonization of national laws and regulations after joining the European Union—but more strict in Southern Europe, including France. Although this may help to explain the relatively favorable trends in countries like France, Spain, and Italy, as has been reported before [54–56], it does not explain our findings for the Nordic countries. Loosening of alcohol control policies coincided with a rise in mortality from alcohol-related causes among lower educated men in Finland, but not in Sweden or Norway, whereas the rise in mortality from alcohol-related causes among lower educated men in Denmark coincided with a slight tightening of alcohol control policies (Figs 3, 4, 7, and 8) [57].

**Fig 11:**
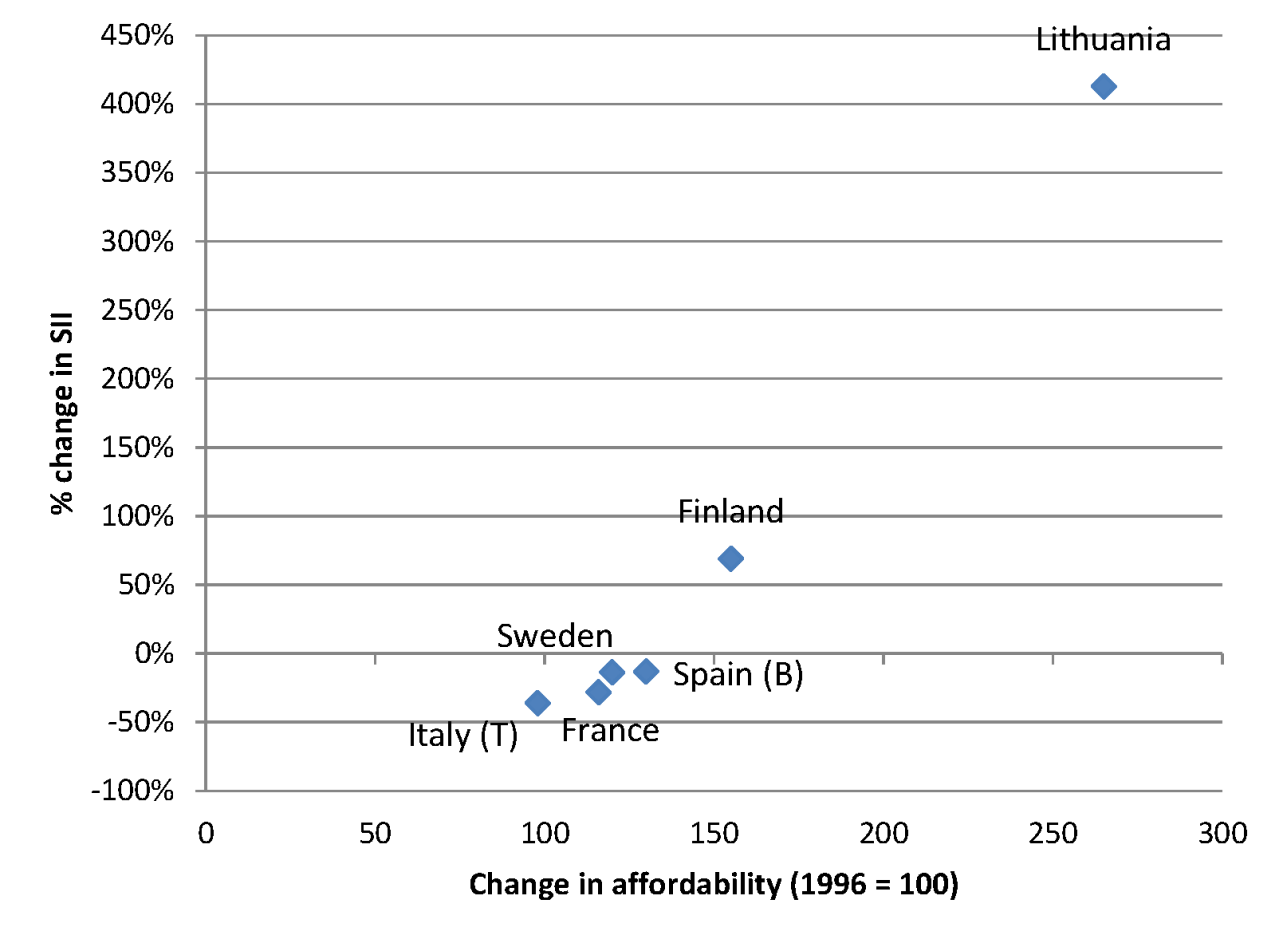
Change in alcohol affordability between 1996 and 2004 versus change in absolute inequality in alcohol-related mortality among men between ca. 1990–1994 and ca. 2005–2009. Data on affordability are from Rabinovich et al. [59] and give levels of affordability relative to that observed in 1996. Only countries with both data on affordability and data on SII for ca. 1990–1994 and ca. 2005–2009 are included in this graph. A change in affordability >100 indicates that, as a result of increasing income and/or decreasing price, alcohol has become more affordable. B, Barcelona; T, Turin.

**Fig 12:**
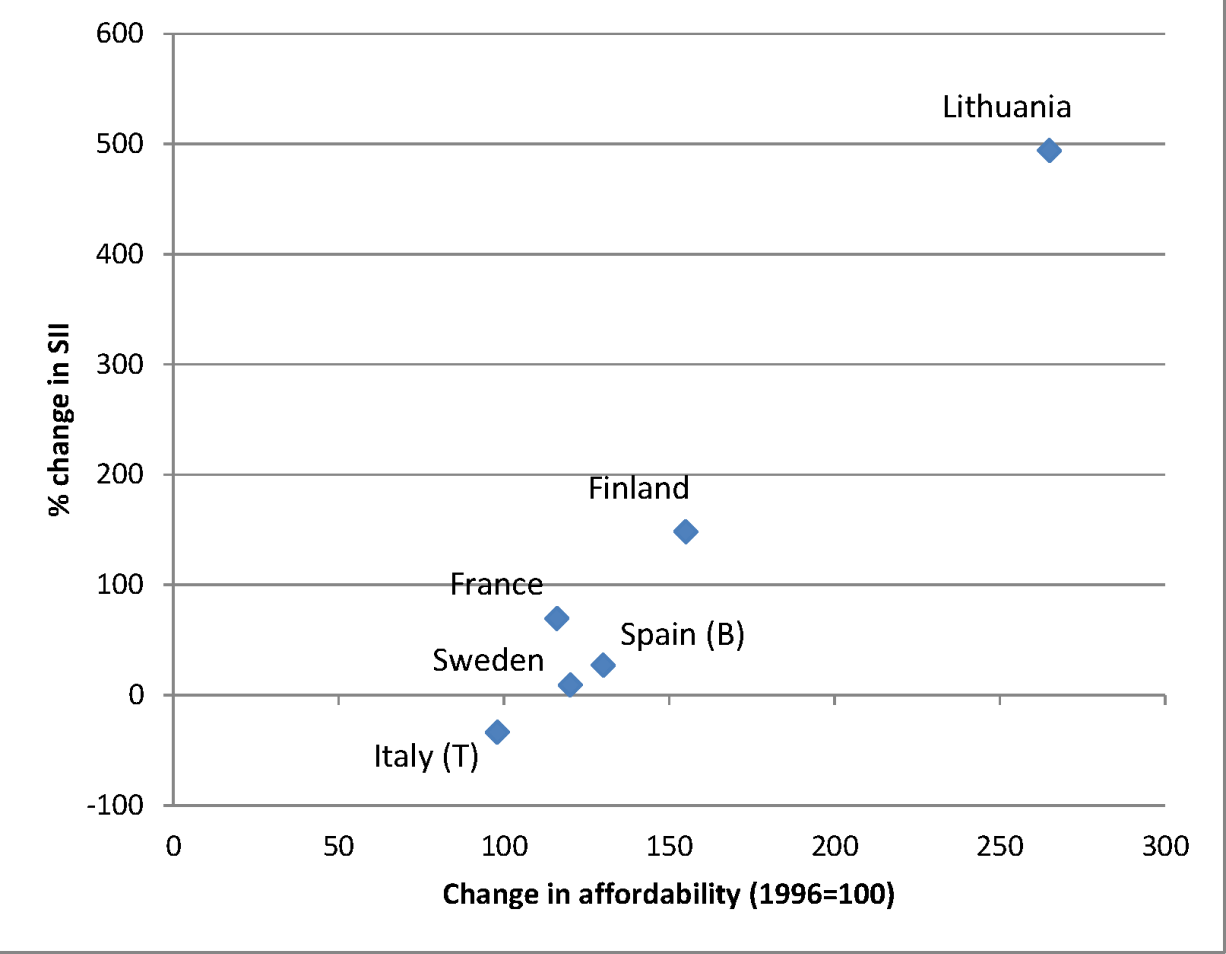
Change in alcohol affordability between 1996 and 2004 versus change in absolute inequality in alcohol-related mortality among women between ca. 1990–1994 and ca. 2005–2009. Data on affordability are from Rabinovich et al. [59] and give levels of affordability relative to that observed in 1996. Only countries with both data on affordability and data on SII for ca. 1990–1994 and ca. 2005–2009 are included in this graph. A change in affordability >100 indicates that, as a result of increasing income and/or decreasing price, alcohol has become more affordable. B, Barcelona; T, Turin.

The increasing affordability of alcohol, due to a combination of rising incomes and stable or sometimes even declining price levels of alcoholic drinks, may also have played a role in the rise of alcohol-related mortality seen in many European countries. Because lower socioeconomic groups are likely to be more responsive to price changes (as shown, for example, in the case of tobacco [58]), the increased affordability of alcohol may have led to relatively larger increases in consumption in lower socioeconomic groups. Between 1996 and 2004, the affordability of alcohol has increased strongly in many European countries, including Lithuania, the United Kingdom, and Finland [59]. Finnish studies have shown that the impact of these changes has been largest in the lowest socioeconomic groups [19,22,23]. Our data suggest that the inequality in alcohol-related mortality has indeed increased most in countries, such as Lithuania and Finland, in which affordability has increased most (Figs 11 and 12).

Trends in alcohol policy in Eastern Europe since 1990 have not been systematically characterized [57]. In many of these countries, excessive alcohol consumption surged around 1990, when cheap alcohol flooded the market [60]. The strong increases of alcohol-related mortality seen in these countries are also likely to be a reflection of the economic crisis following the collapse of the Soviet Union and the resulting stress in the population, and of the extreme liberalization of alcohol markets with a breakdown of control measures in the chaotic first years after the political changes [61–64]. More recently, rising income and increased affordability of alcohol may also have played a role [65].

What can be done to reduce inequalities in alcohol-related mortality? The WHO Commission on Social Determinants of Health has emphasized the importance of tackling the underlying, or “upstream,” causes of inequalities in health, that is, the unequal distribution of socioeconomic resources in society [66]. Some argue that if one focuses efforts to tackle health inequalities on “downstream” causes instead, such as health-related behaviors, the “gap” left by one downstream cause will soon be filled by another, and health inequalities will persist with little change [67]. Although there is some evidence for the exchangeability of downstream causes, such as between smoking and alcohol [21], our between-country comparison shows that it is possible to have small inequalities in alcohol-related mortality with large inequalities in socioeconomic resources, as in Southern Europe, as well as the reverse, as in Northern Europe.

This implies that policies and interventions redressing the higher prevalence of harmful alcohol consumption patterns in lower socioeconomic groups could help to reduce inequalities in mortality, even without tackling the “upstream” causes of health inequalities. The evidence base shows that a range of alcohol control measures are cost-effective [68]. Although little is known about differential effectiveness by socioeconomic position [69], an informed guess is that a combination of different approaches will be necessary. Making alcohol more expensive has been shown to be the most cost-effective way to reduce alcohol-related harm, and will probably also work to reduce the inequality in alcohol-related mortality. Whatever the policies and interventions chosen, however, they should take into account cultural preferences regarding drinking and should include measures to control illegal trade and production.

### Conclusions

Alcohol-related conditions play an important role in generating socioeconomic inequality in total mortality in many European countries. Countering increases in alcohol-related mortality in lower socioeconomic groups is essential for reducing inequalities in mortality. Studies of why such increases have not occurred in some countries, such as France, Switzerland, Spain, and Italy, can help in developing evidence-based policies in other European countries.

## Supporting Information

S1 STROBE Checklist (DOC)

S1 Fig Trends in age-adjusted mortality from alcohol-related causes among men aged 35–64 y with non-manual occupations, ca. 1980–2010, by population. (TIF)

S2 Fig Trends in age-adjusted mortality from alcohol-related causes among men aged 35–64 y with manual occupations, ca. 1980–2010, by population. (TIF)

S1 Table Data sources: quantitative characteristics for latest observed period among men, by education level. For the time periods to which these data apply, see Table 2. Percentage population by education level was calculated among those with known education level.
(XLSX)

S2 Table Data sources: quantitative characteristics for latest observed period among men, by occupational class. For the time periods to which these data apply, see Table 2. Percentage population by occupational class was calculated among those with known occupational class.
(XLSX)

S3 Table Data sources: quantitative characteristics for latest observed period among women, by education level. For the time periods to which these data apply, see Table 2. Percentage population by education level was calculated among those with known education level.
(XLSX)

S4 Table ICD code numbers. (XLSX)

S5 Table Age-adjusted rate ratio and rate difference for occupational class inequality in mortality from alcohol-related causes in the most recent observed period, by population, for men aged 35–64 y. RD, rate difference; RR, rate ratio.
(XLSX)

S6 Table Trends in the rate ratio of relative occupational class inequality in mortality from alcohol-related causes, by population, for men aged 35–64 y. For Basque Country and Estonia, no data on occupational class inequality in mortality from alcohol-related causes were available for periods earlier than those presented in S5 Table.
(XLSX)

S7 Table Trends in the rate difference of absolute occupational class inequality in mortality from alcohol-related causes, by population, for men aged 35–64 y. For Basque Country and Estonia, no data on occupational class inequality in mortality from alcohol-related causes were available for periods earlier than those presented in S5 Table.
(XLSX)

## Abbreviations

RII: relative index of inequality
SII: slope index of inequality
