# Supplementary material for: Inequalities in Alcohol-Related Mortality in 17 European Countries: A Retrospective Analysis of Mortality Registers
Source: PLoS Med. 2015 Dec 1;12(12):e1001909. doi: 10.1371/journal.pmed.1001909 (PMC4666661; doi:10.1371/journal.pmed.1001909)
Supplement: S1 STROBE Checklist — (DOC) [file pmed.1001909.s001.doc]

STROBE Statement—checklist of items that should be included in reports of observational studies

**Inequalities in alcohol-related mortality in 16 European countries**

|  | Item No | Recommendation |
| --- | --- | --- |
| **Title and abstract** | 1 | (*a*) Indicate the study’s design with a commonly used term in the title or the abstract  **Abstract: “a retrospective analysis of mortality registers”** |
|  |  | (*b*) Provide in the abstract an informative and balanced summary of what was done and what was found  **Done** |
| Introduction | | |
| Background/rationale | 2 | Explain the scientific background and rationale for the investigation being reported  **The introduction is devoted completely to explaining this background and rationale** |
| Objectives | 3 | State specific objectives, including any prespecified hypotheses  **Study questions: “we set out to study whether European countries differ in the magnitude of socioeconomic inequalities in alcohol-related mortality, and what the trends of these inequalities over the past decades have been”** |
| Methods | | |
| Study design | 4 | Present key elements of study design early in the paper  **Data and methods, first paragraph of Data subsection:** “**Data come from ….”** |
| Setting | 5 | Describe the setting, locations, and relevant dates, including periods of recruitment, exposure, follow-up, and data collection  **Web appendix table A1** |
| Participants | 6 | (*a*) *Cohort study*—Give the eligibility criteria, and the sources and methods of selection of participants. Describe methods of follow-up  **Data and methods, first paragraph of Data subsection:** “**Data come from ….”** |
|  |  | (*b*) *Cohort study*—For matched studies, give matching criteria and number of exposed and unexposed  **N/A** |
| Variables | 7 | Clearly define all outcomes, exposures, predictors, potential confounders, and effect modifiers. Give diagnostic criteria, if applicable  **Data and methods, second and third paragraphs of Data subsection, and second paragraph of Analysis subsection: “We use two indicators of …”, “Our main outcome variable, mortality due to …”, “…controlling for age as a dummy variable (in 5-year age groups)”.** |
| Data sources/ measurement | 8* | For each variable of interest, give sources of data and details of methods of assessment (measurement). Describe comparability of assessment methods if there is more than one group  **Data and methods, first second and third paragraphs of Data subsection. Comparability issues are discussed in the Discussion, second third and fourth paragraph of Strengths and limitations subsection: “It has been suggested that …”, “Although under-recording is …”, “Over- and under-recording may also differ between …”.** |
| Bias | 9 | Describe any efforts to address potential sources of bias  **Data and methods, second paragraph of Analysis subsection: “We study the magnitude of both relative and absolute inequalities, using the Relative Index of Inequality (RII) and the Slope Index of Inequality (SII) …”** |
| Study size | 10 | Explain how the study size was arrived at  **We study complete national or regional populations.** |
| Quantitative variables | 11 | Explain how quantitative variables were handled in the analyses. If applicable, describe which groupings were chosen and why  **Data and methods, second paragraph of Data subsection and first and second paragraph of Analysis subsection: “We use two indicators of socioeconomic position …”, “Our analysis consists of …”, “We study the magnitude of …”** |
| Statistical methods | 12 | (*a*) Describe all statistical methods, including those used to control for confounding  **Data and methods, first and second paragraph of Analysis subsection: “Our analysis consists of …”, “We study the magnitude of …”** |
|  |  | (*b*) Describe any methods used to examine subgroups and interactions  **N/A** |
|  |  | (*c*) Explain how missing data were addressed  **Web appendix table A1.** |
|  |  | (*d*) *Cohort study*—If applicable, explain how loss to follow-up was addressed  **Data and methods, first paragraph of Data subsection:** “**Data come from ….”** |
|  |  | (*e*) Describe any sensitivity analyses **N/A** |

| Results | | |
| --- | --- | --- |
| Participants | 13* | (a) Report numbers of individuals at each stage of study—eg numbers potentially eligible, examined for eligibility, confirmed eligible, included in the study, completing follow-up, and analysed **N/A** |
|  |  | (b) Give reasons for non-participation at each stage **N/A** |
|  |  | (c) Consider use of a flow diagram **N/A** |
| Descriptive data | 14* | (a) Give characteristics of study participants (eg demographic, clinical, social) and information on exposures and potential confounders  **Because of the overwhelming amount of material in our paper, we have decided not to separately present characteristics of study participants.** |
|  |  | (b) Indicate number of participants with missing data for each variable of interest  **Web appendix table A1.** |
|  |  | (c) *Cohort study*—Summarise follow-up time (eg, average and total amount)  **Web appendix table A1.** |
| Outcome data | 15* | *Cohort study*—Report numbers of outcome events or summary measures over time  **Web appendix table A1.** |
|  |  |  |
|  |  |  |
| Main results | 16 | (*a*) Give unadjusted estimates and, if applicable, confounder-adjusted estimates and their precision (eg, 95% confidence interval). Make clear which confounders were adjusted for and why they were included **In this case, reporting non-adjusted estimates (i.e., estimates not adjusted for age) does not make much sense. The adjustment for age has been reported wherever relevant, e.g. title of figures 1 and 2** |
|  |  | (*b*) Report category boundaries when continuous variables were categorized **N/A** |
|  |  | (*c*) If relevant, consider translating estimates of relative risk into absolute risk for a meaningful time period  **We have systematically reported both relative and absolute effect measures (e.g. in table 1 and table 2, where we report both RII and SII).** |
| Other analyses | 17 | Report other analyses done—eg analyses of subgroups and interactions, and sensitivity analyses  **N/A** |
| Discussion | | |
| Key results | 18 | Summarise key results with reference to study objectives **This is the main focus of the Discussion, Summary of main findings subsection** |
| Limitations | 19 | Discuss limitations of the study, taking into account sources of potential bias or imprecision. Discuss both direction and magnitude of any potential bias **This is the main focus of the Discussion, Strengths and limitations subsection** |
| Interpretation | 20 | Give a cautious overall interpretation of results considering objectives, limitations, multiplicity of analyses, results from similar studies, and other relevant evidence **This is the main focus of the Discussion, Interpretation subsection** |
| Generalisability | 21 | Discuss the generalisability (external validity) of the study results **As our study covers complete national or regional populations, this is not applicable**. |
| Other information | | |
| Funding | 22 | Give the source of funding and the role of the funders for the present study and, if applicable, for the original study on which the present article is based **Acknowledgement section: “Supported by a grant …”** |
