# Supplementary figures and images for: Inequalities in Alcohol-Related Mortality in 17 European Countries: A Retrospective Analysis of Mortality Registers
Source: PLoS Med. 2015 Dec 1;12(12):e1001909. doi: 10.1371/journal.pmed.1001909 (PMC4666661; doi:10.1371/journal.pmed.1001909)

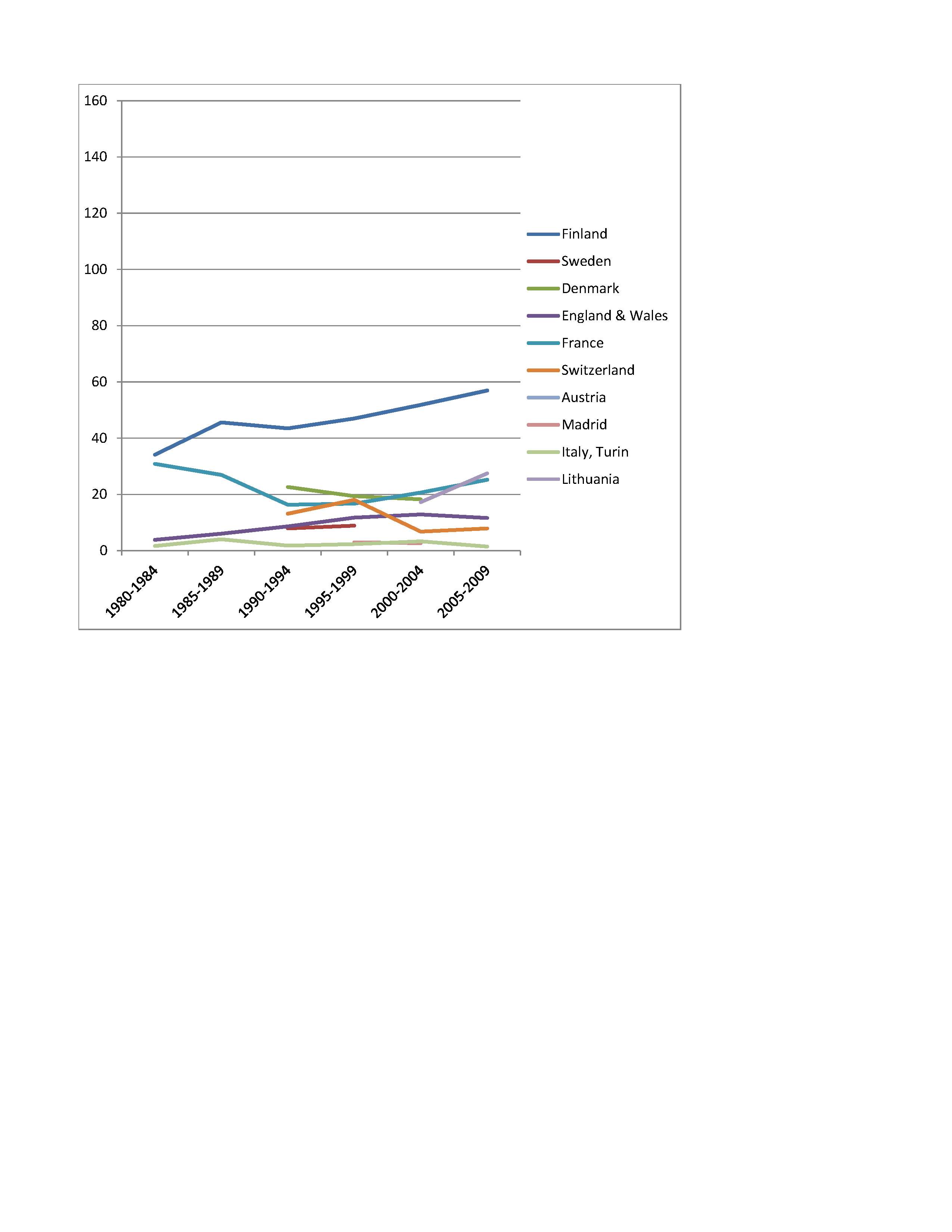

Supplement: S1 Fig — (TIF) [file pmed.1001909.s002.tif]

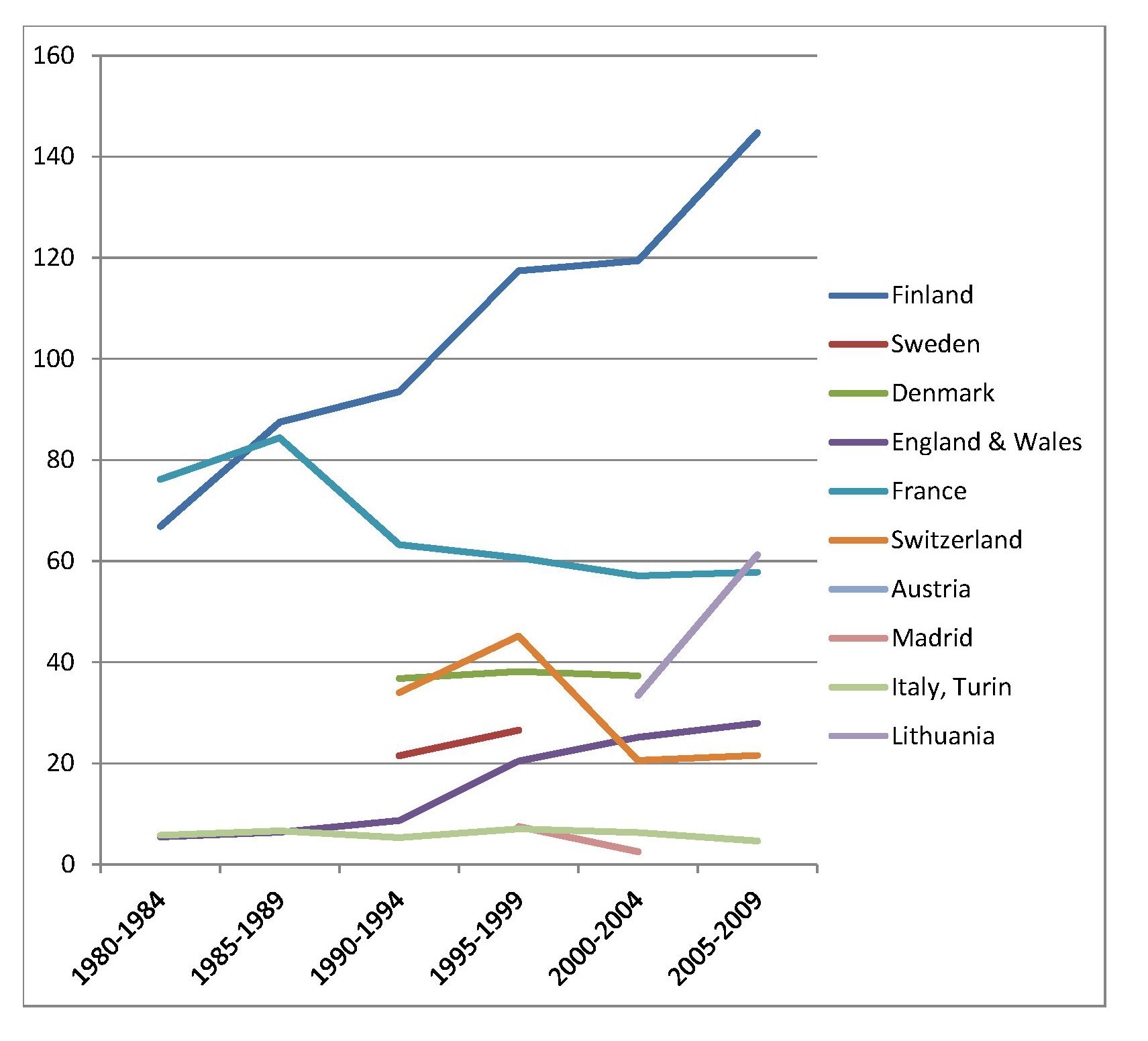

Supplement: S2 Fig — (TIF) [file pmed.1001909.s003.tif]
